# Evolutionary diversification of the HAP2 membrane insertion motifs to drive gamete fusion across eukaryotes

**DOI:** 10.1371/journal.pbio.2006357

**Published:** 2018-08-13

**Authors:** Juliette Fedry, Jennifer Forcina, Pierre Legrand, Gérard Péhau-Arnaudet, Ahmed Haouz, Mark Johnson, Felix A. Rey, Thomas Krey

**Affiliations:** 1 Unité de Virologie Structurale, Institut Pasteur, Paris, France; 2 CNRS UMR 3569, Paris, France; 3 Universite Paris Descartes Sorbonne Paris Cité, Institut Pasteur, Paris, France; 4 Institute of Virology, Hannover Medical School, Hannover, Germany; 5 Brown University, Department of Molecular Biology, Cell Biology, and Biochemistry, Providence, Rhode Island, United States of America; 6 Synchrotron SOLEIL, L'Orme des Merisiers, Gif-sur-Yvette, France; 7 Ultrapole, CNRS UMR 3528, Institut Pasteur, Paris, France; 8 Protéopôle, CNRS UMR 3528, Institut Pasteur, Paris, France; 9 German Center for Infection Research (DZIF), Hannover, Germany; National Institutes of Health, United States of America

## Abstract

HAPLESS2 (HAP2) is a broadly conserved, gamete-expressed transmembrane protein that was shown recently to be structurally homologous to viral class II fusion proteins, which initiate fusion with host cells via insertion of fusion loops into the host membrane. However, the functional conformation of the HAP2 fusion loops has remained unknown, as the reported X-ray structure of *Chlamydomonas reinhardtii* HAP2 lacked this critical region. Here, we report a structure-guided alignment that reveals diversification of the proposed HAP2 fusion loops. Representative crystal structures show that in flowering plants, HAP2 has a single prominent fusion loop projecting an amphipathic helix at its apex, while in trypanosomes, three small nonpolar loops of HAP2 are poised to interact with the target membrane. A detailed structure-function analysis of the *Arabidopsis* HAP2 amphipathic fusion helix defines key residues that are essential for membrane insertion and for gamete fusion. Our study suggests that HAP2 may have evolved multiple modes of membrane insertion to accommodate the diversity of membrane environments it has encountered during eukaryotic evolution.

## Author summary

The fusion of gamete plasma membranes is the fundamental cellular event that brings two parental cells together to form a new individual, yet we know surprisingly little about this process at the molecular level. HAPLESS 2 (HAP2) is a conserved sperm plasma membrane protein that is essential for gamete fusion in a diverse array of eukaryotes. It was recently shown to share a common ancestor with viral proteins that drive fusion of the viral envelope with host membranes, but its mechanism of action remained elusive, since the reported structure did not resolve the proposed membrane interaction surface. Here, we report two new HAP2 structures revealing that HAP2 has evolved diverse membrane interaction surfaces. In the flowering plants, HAP2 uses an amphipathic helix that presents nonpolar residues to the target membrane; in trypanosomes, the membrane interaction surface comprises three shallow nonpolar loops.

## Introduction

The fusion of gamete plasma membranes to form a zygote is central to sexual reproduction, yet a molecular mechanism for this fundamental process has only very recently been proposed. The crystal structure of the *C*. *reinhardtii* HAPLESS 2 (HAP2) ectodomain (*Cr*HAP2e) [1] revealed that this broadly conserved, gamete-expressed transmembrane protein [2–4] has the same three-dimensional fold as class II viral fusion proteins. It was proposed that, like its viral counterparts, HAP2 initiates gamete fusion by insertion of fusion loops into the opposing gamete plasma membrane [5]. Consistent with this idea, mutations in the proposed fusion loops of *Cr*HAP2e disrupted its ability to insert into artificial membranes in vitro and mediate gamete fusion in vivo [1]. Because the reported *Cr*HAP2e structure did not include the critical region of the fusion loops, the question of how HAP2 inserts into the opposing gamete plasma membrane was left unanswered.

Viral fusion proteins have been divided into several structural classes that use either fusion peptides (class I) or fusion loops (classes II and III) to insert into host cell membranes [6]. HAP2 is homologous to the viral class II fusion proteins, which fold into an articulated rod made of three β-sheet-rich domains, termed I, II, and III, with the central domain I flanked by domains II and III in the prefusion form [7–9]. Domain III connects to the C-terminal transmembrane domain via a flexible linker, while domain II bears the fusion loops at the opposite end.

Stable insertion into the host membrane is required to withstand pulling forces that occur as the fusion protein bridges the intermembrane space via a transient extended intermediate state that later collapses into a trimeric “hairpin” conformation, which drives the merger of the viral envelope and host membrane [10]. The interaction with membranes was best studied for the class I proteins, in which the fusion peptide is an N-terminal extension that folds independently of the rest of the fusion protein upon its insertion into a lipid bilayer, exposing a relatively extensive nonpolar platform to the outer leaflet [11]. In the case of the influenza virus hemagglutinin, the prototypic class I fusion protein, it was shown that the fusion peptide forms an α-helical hairpin [12], exposing nine bulky nonpolar side chains to the external lipid layer of the host membrane. In contrast, class II proteins from arthropod-borne viruses (arboviruses) were proposed to insert internal fusion loops located at the domain II “tip,” i.e., at the end of the protein opposite the C-terminal transmembrane domain integral to the viral envelope. In contrast to the class I proteins, the fusion loops do not change conformation upon interaction with lipids, and the fusion protein of the Rift Valley fever virus (RVFV) was shown to feature an internal pocket that specifically accommodates the head group of glycerophospholipids from the outer leaflet of the target membrane [13]. The resulting polar interaction network allows stable insertion of the protein with only one or two bulky aromatic side chains of the fusion loops exposed to the aliphatic moiety of the membrane. Understanding how HAP2 achieves stable insertion into the target membrane is key for understanding the molecular mechanism driving gamete fusion. The diversity of reproductive systems in which HAP2 has been implicated across eukaryotic organisms likely represents a large variety of fusion environments (e.g., lipid compositions of gamete membranes), and it remains elusive whether HAP2 has evolved different membrane interaction surfaces to secure the initial interaction with the target membrane.

Here, we carried out a comparative structure/function study of HAP2 from distantly related eukaryotes and found striking sequence variability at the domain II tip, which features multiple insertions and deletions, contrasting with the relative conservation of the rest of the protein. We then obtained structural data for HAP2 from two organisms that displayed the most contrasting diversity in this region: the flowering plant *Arabidopsis thaliana* (*At*HAP2) and the protozoan *Trypanosoma cruzi* (*Tc*HAP2). The X-ray structures confirmed that the membrane interaction surfaces were totally different: while in *At*HAP2 there is a single fusion loop that projects an amphipathic helix (termed αF) toward the membrane (an unprecedented feature in viral class II fusion proteins), in *Tc*HAP2, the membrane interaction surface is composed of three short loops. Biochemical and genetic experiments focusing on αF confirmed that the nonpolar residues are required for membrane insertion in vitro and for gamete fusion in vivo. Bioinformatic analyses show that αF is likely to be conserved across flowering plants, and functional studies revealed that it is interchangeable between rice and *Arabidopsis*, which are among the most distantly related flowering plants.

## Results

### Sequences encoding the proposed HAP2 fusion loops are highly divergent

**Fig 1:**
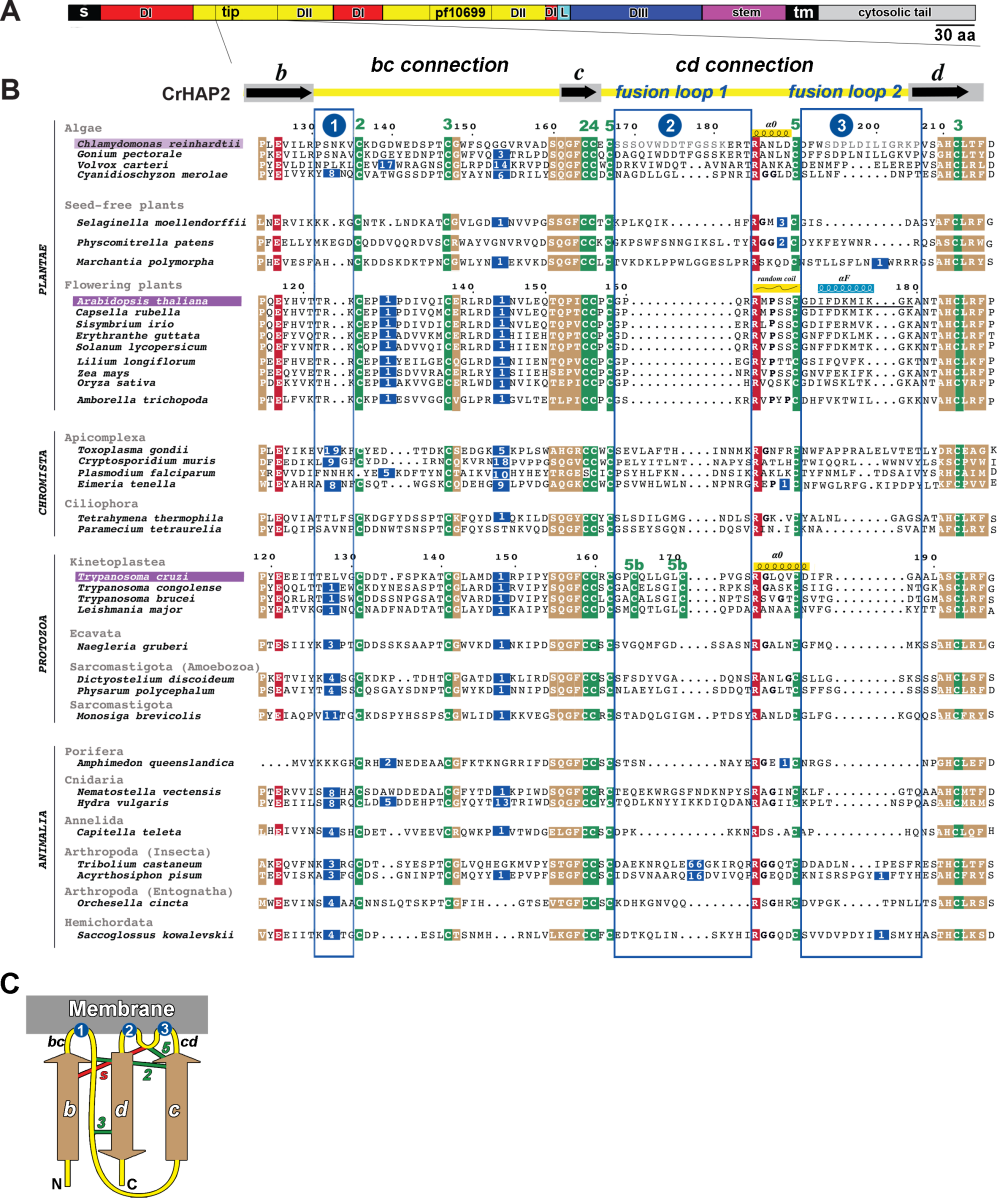
Substantial divergence in the domain II tip of HAP2 across eukaryotes. (A) Linear diagram of the HAP2 primary structure indicating the positions of the signal peptide (“S”), the polypeptide segments corresponding to domains I, II, and III (“DI–III”), highlighting the tip of domain II (the *bdc* β-sheet, aligned in B), the pfam HAP2-GCS1 domain (pf10699), the linker (“L”) between DI and DIII, the stem that connects the ectodomain to the transmembrane domain (“tm”), and the cytosolic tail. (B) HAP2 aa sequence alignment obtained with MUSCLE [16] using representative members from four out of the five eukaryotic kingdoms [17] named on the left column. *A*. *thaliana* and *T*. *cruzi*, the two orthologs studied further in this work, are highlighted in purple background, as well as *C*. *reinhardtii*, which served as reference. Only the *bdc* β-sheet region is displayed, with black arrows marking the β-stands in the linear diagram drawn above, and the intervening connections in yellow (*bc* and *cd*) [1] with the putative *Cr*HAP2 fusion loops 1 and 2 in the *cd* connection labeled. In the alignment, conserved and semiconserved residues are highlighted on red and beige backgrounds, respectively. A green background highlights cysteine residues, most of which are strictly conserved, involved in disulfide bonds numbered above the alignment according to the *Cr*HAP2 structure. For clarity, insertions with respect to *Cr*HAP2 are omitted, but their length is indicated on a dark blue background; the three major insertion/deletion hotspots in regions potentially interacting with the target membrane are framed in blue and numbered above the alignment in blue circles. The *Cr*HAP2 α0 helix separating loops 1 and 2 is indicated with a helical symbol immediately above the sequence. Potential helix-breaking residues (glycine or proline) in the α0 region are indicated in bold characters. A random coil above the *A*. *thaliana* sequence highlights the absence of the α0 helix, and a helical symbol on a cyan background indicates the position of the *A*. *thaliana* fusion helix αF described in the text (Figs 2 and 3). A black underline marks the *A*. *thaliana* segment deleted in the *At*ΔαF construct used in genetic assays (see below, section αF amphipathicity is required for *At*HAP2 in vitro liposome insertion and in vivo function). (C) Schematic diagram of the *bdc* β-sheet and the connecting loops at the tip of domain II relative to the target membrane. The β-strands are colored beige to match the conserved blocks in the alignment. Drawn are the disulfide bonds (green bars, numbered as in the alignment) and the salt bridge (red bar, labeled “s”), which are conserved features constraining the organization of the domain II tip. Blue circles indicate the position of insertion hotspots (marked identically in B at the top of the alignment) predicted to project residues into the target membrane for insertion, the first one in the *bc* β-strands connection (left) and the two others in the *cd* connection (right). aa, amino acid; *Cr*HAP2, *C*. *reinhardtii* HAP2; HAP2, HAPLESS 2

The domain II tip is the region of class II fusion proteins that must firmly insert into target membranes to initiate the fusion process. In order to probe the potential structural diversity of HAP2 in this region, we used the structure of *Cr*HAP2e to guide the alignment of 38 HAP2 amino acid (aa) sequences representing organisms from four eukaryotic kingdoms (Fig 1, S1 Table). We focused on the region corresponding to the *b*, *c*, and *d* β-strands and their interstrand connections at the domain II tip (Fig 1), which contains the fusion loops [1] (Fig 1B). Nine invariant residues anchored the alignment: seven cysteines (in green background) participating in highly conserved disulfide bonds; a conserved glutamate in β-strand *b*; and a conserved arginine residue in the *cd* connection, making a salt-bridge with the conserved glutamate, both residues shown in red background in Fig 1B (see Materials and methods). The resulting alignment showed, as expected, that sequences from relatively closely related groups (e.g., flowering plants) were similar to each other. But the comparison between different phylogenetic groups revealed three regions (vertical blue frames, Fig 1B) with high diversity in length and primary sequence right at the tip of domain II: regions 2 and 3 correspond to the two *At*HAP2 predicted fusion loops [1] in the connection between strands *c* and *d*. Variable region 1 is at the beginning of the long *bc* connection, immediately downstream of strand *b* and preceding disulfide 2 at the membrane-proximal end of the *bdc* β-sheet (Fig 1C). Residues within this region of the *bc* connection were indeed shown to be part of the membrane-interacting region of other class II fusion proteins, e.g., bunyavirus Gc [14, 15]. Although variable region 1 is very short and retracted in *Cr*HAP2 [1], and residues from this region are not predicted to reach the target membrane, the sequence alignment indicates that this is not the case in HAP2 from other organisms. For example, several orthologs have an insertion (left-most vertical blue box, variable region 1, Fig 1B)—the most prominent in the apicomplexan parasite *Toxoplasma gondii*—potentially making a loop projecting apically to make contact with the target membrane.

The HAP2 segment predicted to form fusion loop 1 in *Chlamydomonas* is highly variable (blue frame 2 in Fig 1B). This region is absent in flowering plants, displays an additional and unique pair of cysteine residues expected to make an extra disulfide bond in kinetoplastids, and is much larger in insects (66-residue insertion in *Tribolium castaneum*). The alignment also shows that most orthologs have significant deletions in the variable region 3 (third blue frame, Fig 1B), as observed in protozoan and animal sequences from the Porifera, Cnidaria, and Annelida. The short segment corresponding to the *Cr*HAP2 α0 helix varies in length from four to seven residues and features α-helix-breaking residues (glycine or proline) in several orthologs (Fig 1B, in between the second and third blue boxes), suggesting α0 may not be a conserved structural feature of HAP2. The alignment of the predicted HAP2 membrane interaction domain from species representing the eukaryotic diversity therefore suggests that HAP2 has evolved multiple structural motifs for insertion into the target membrane.

### The HAP2 core structure is conserved across eukaryotes

To understand the organization of the divergent structural motifs used by HAP2 for membrane insertion, we selected representative sequences for further study from the flowering plants and the kinetoplastids, which were among those with the most contrasting features in variable regions 2 and 3 (Fig 1B). We expressed the recombinant ectodomains of *A*. *thaliana* HAP2 (*At*HAP2e; aa 25–494) and *T*. *cruzi* HAP2 (*Tc*HAP2e, aa 26–516) in *Drosophila* Schneider 2 (S2) cells for structural studies (see Materials and methods). Size exclusion chromatography (SEC) and multiangle static light scattering (MALS) revealed that both proteins behaved as monomers in solution (S1A–S1D Fig). *At*HAP2e crystallized in the P6_3_ hexagonal space group and diffracted anisotropically to a mean nominal resolution of 2.75 Å. *Tc*HAP2e only crystallized upon limited proteolysis using subtilisin (see Materials and methods). The resulting purified protease-resistant 40-kD fragment (termed *Tc*HAP2e_sub_, S1E Fig) crystallized in the hexagonal space group P6_1_22 (S1F and S1G Fig), and the best crystals diffracted to 3.1-Å resolution (see Materials and methods; crystallographic statistics are listed in S2 Table). We determined both structures by the molecular replacement method using a search model derived from the structure of *C*rHAP2e (Protein Data Bank [PDB]: 5MF1)

**Fig 2:**
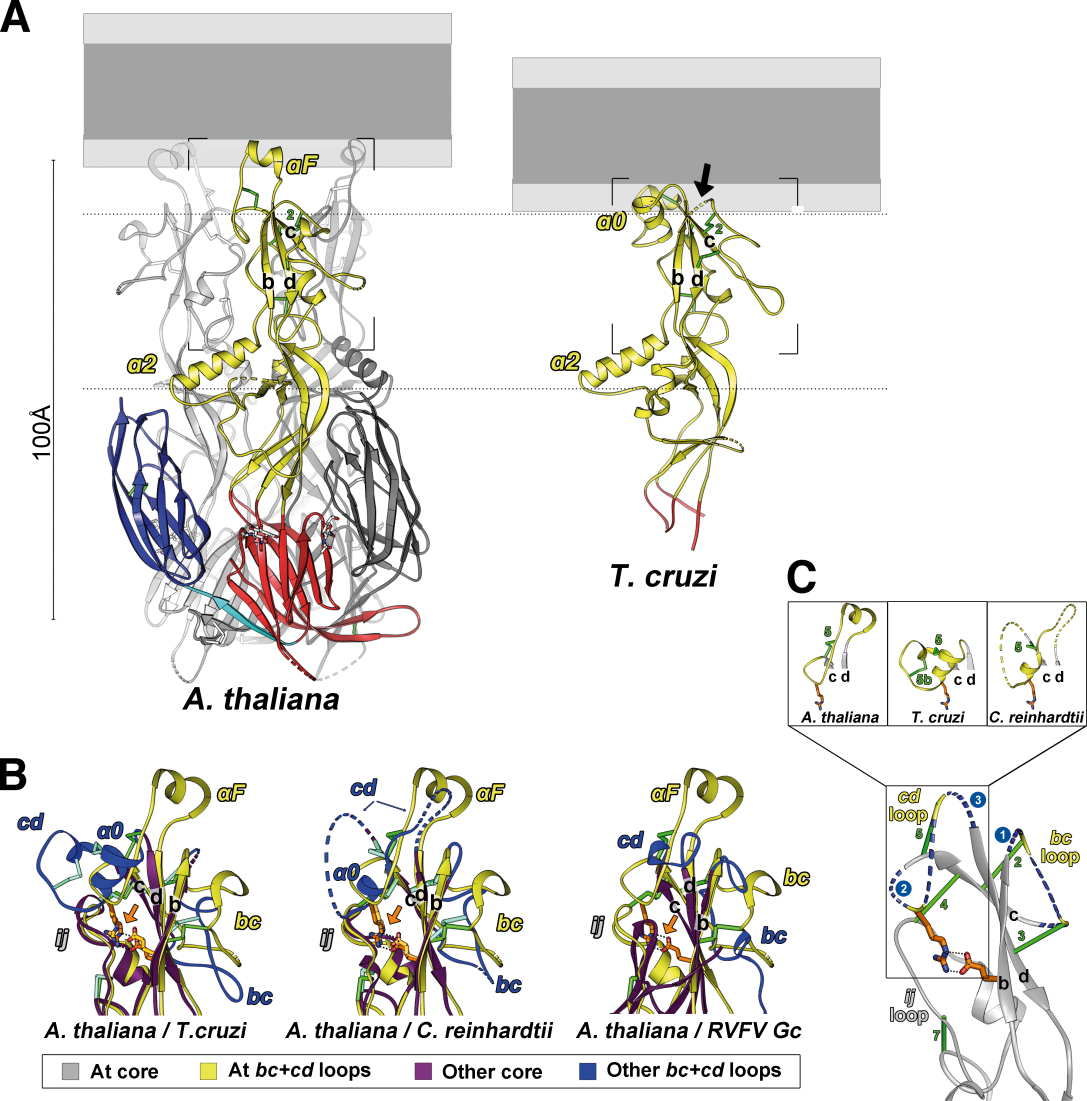
Structures of *A*. *thaliana* and *T*. *cruzi* HAP2. (A) Ribbon representation of the *At*HAP2 trimer (left panel) and domain II of *Tc*HAP2 (right). A “fused” membrane bilayer is diagrammed above, roughly to scale and with light and dark gray indicating polar and nonpolar moieties, respectively, to illustrate the relation of the fusion loops with respect to a membrane upon insertion. The *At*HAP2 trimer subunit in the foreground and the *Tc*HAP2 monomeric domain II are colored according to the class II convention: red, yellow, and blue for domains I, II, and III, respectively; cyan for the domain I–III linker. Disulfide bonds are shown as green sticks, and dashed tubes indicate disordered regions in the crystal (including in the membrane interaction surface in *Tc*HAP2 domain II, arrow on the right panel) (B) Pairwise superposition of the *At*HAP2 (in yellow) on *Tc*HAP2, *Cr*HAP2, and RVFV Gc [13]. The core *bdc* β-sheet and *ij* loop of the latter are drawn in purple, with connecting loops in blue, to highlight the structurally conserved domain II scaffold. The conserved disulfide bonds (green) and salt bridge (colored according to atom type) are shown as sticks, hydrogen bonds as dotted lines. An orange arrow highlights the striking superposition of the side chains of the conserved salt bridge, as only the Cα atoms of the β-strands were used for the superposition. (C) Cartoon representation of the structurally conserved scaffold (in gray) in the HAP2 tip region, with variable loops in yellow. The three insets show the observed conformations of the variable *cd* connection in the known structures (*At*, *Tc*, and *Cr*HAP2). *At*HAP2, *A*. *thaliana* HAP2; *Cr*HAP2, *C*. *reinhardtii* HAP2; HAP2, HAPLESS 2; RVFV, Rift Valley fever virus; *Tc*HAP2, *T*. *cruzi* HAP2

**Fig 3:**
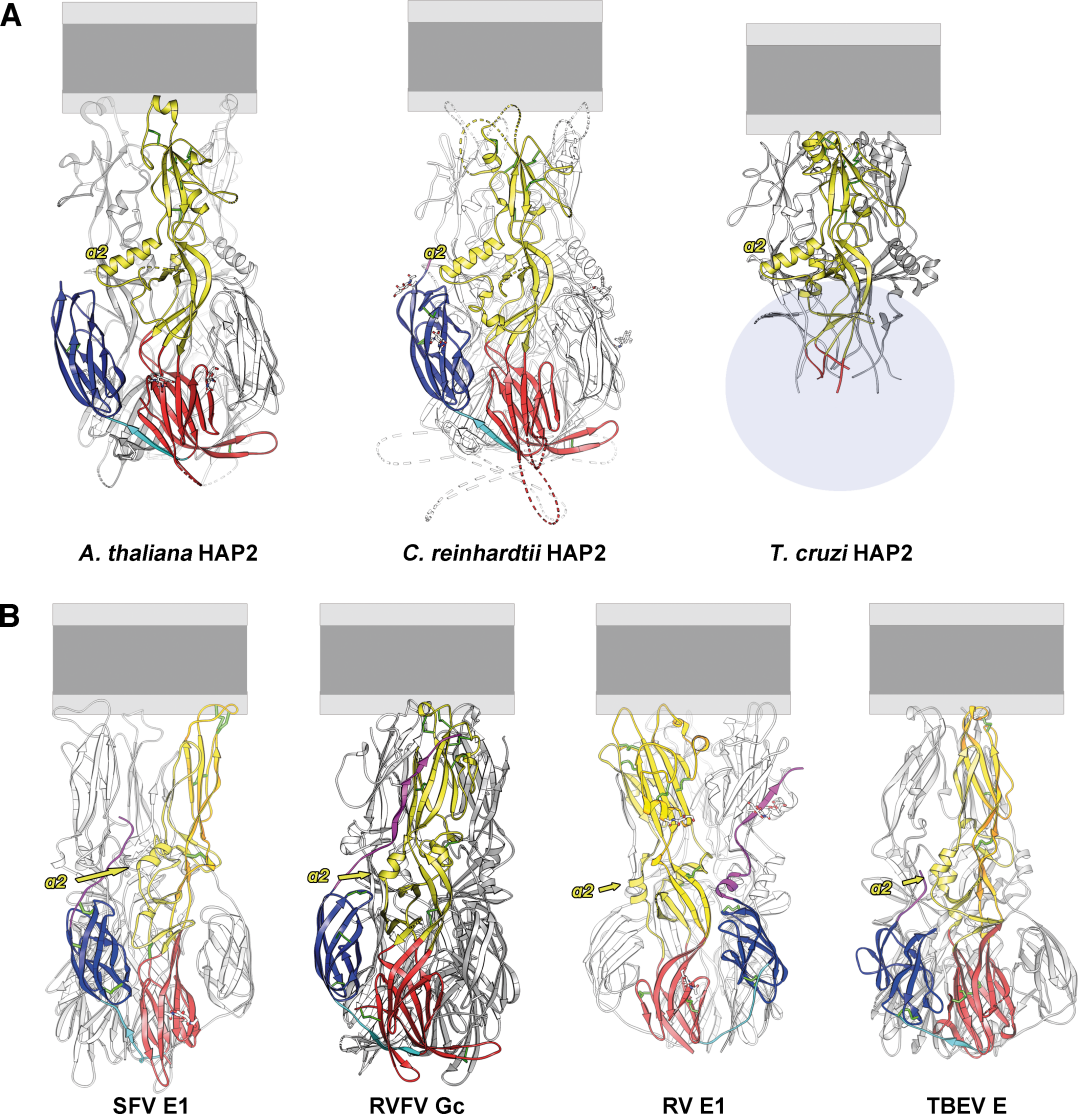
Side-to-side comparison of the HAP2 structures and relation to viral class II proteins. (A) The postfusion trimer structures of *At*HAP2e and *Cr*HAP2e (PDB 5MF1;[1]) and the model for a *Tc*HAP2 trimer obtained by superimposing the domain II structure on the *Cr*HAP2e trimer. In the third panel, a grey oval indicates the region expected to be occupied by domains I and III of *Tc*HAP2, for which we do not have a structure, but for which the aa sequence alignment indicates they are very closed, except for some inter β-strand connections. (B) The corresponding postfusion trimer structures of four representative viral class II fusion proteins: SFV E1 (PDB 1RER; [18]), RVFV Gc (PDB 6EGU; [13]), RV E1 (PDB 4ADI; [19]) and TBEV E (PDB 1URZ; [20]). The structures show the same organization of the three domains (yellow, red, and blue), which also share the same fold. Note that as expected, the viral proteins have evolved further from each other, as the same secondary structure elements are present, but their relative orientations have changed. The HAP2 structures can be considered as resembling a “frozen” ancestor of the class II fusion proteins observed on present-day viruses, given the huge difference in generation time and in the rate of mutations of RNA virus replication when compared with eukaryotic organisms. The statistics obtained with the DALI server [21] from the comparison of domain II from the various structures are provided in S3 Table. The HAP2 orthologs can be aligned with respect to the membrane using the highly conserved α2-helix. In contrast, the same helix does not display a conserved orientation and length in viral class II fusion proteins and therefore does not allow a similar alignment. *At*HAP2e, *A*. *thaliana* HAPLESS 2 ectodomain; *Cr*HAP2e, *C*. *reinhardtii* HAPLESS 2 ectodomain; HAP2, HAPLESS 2; PDB, Protein Data Bank; RV, rubella virus; RFVF, Rift Valley fever virus; SFV, Semliki Forest virus; TBEV, tick-borne encephalitis virus; *Tc*HAP2, *T*. *cruzi* HAPLESS 2

The experimental electron density map of *At*HAP2 allowed us to trace 446 out of 469 ectodomain residues, including the region at the tip of domain II (S1H Fig, right panel) that was disordered in *Cr*HAP2. The resulting atomic model of *At*HAP2 revealed a trimer in unequivocal postfusion hairpin conformation (Figs 2A and 3A), as observed previously for *Cr*HAP2. The crystals of *Tc*HAP2e_sub_ consisted of domain II with a short extension into domain I (Fig 2A). There was continuous electron density for the loops at the tip of domain II (S1H Fig, left panel), and we could build the polypeptide chain unambiguously in this region, albeit for a 2-residue break immediately after strand *b* (i.e., in the *bc* loop, Fig 2A). Monomeric *Tc*HAP2e_sub_ domain II displayed the same conformation adopted by domain II in the trimers of both *At*HAP2e and *Cr*HAP2e (Fig 2A). We were thus able to superpose the *TcHAP2* domain II on its counterparts of either the *Cr*HAP2 or the *At*HAP2 trimer with reasonable subunit packing, suggesting that the postfusion *TcHAP2* trimer would look very similar (Fig 3A).

The prominent α2 helix in domain II is nearly identical in length and orientation between *At*HAP2, *Tc*HAP2, and *Cr*HAP2 (Fig 2A and Fig 3) and thus appears as a HAP2 structural hallmark. Pairwise comparisons of domain II from the three available HAP2 structures indeed showed very high DALI scores [21], relative to a similar comparison between viral class II proteins (S3 Table). To further compare HAP2 to its viral counterparts, we superposed the *bdc* β-sheet of domain II. The root-mean-square deviation (rmsd) obtained when superimposing the core 19 Cα atoms of the HAP2 *bdc* β-sheet onto the corresponding element in the flavivirus, alphavirus, and bunyavirus class II proteins ranged between 2.3 and 2.7 Å. Pairwise comparisons of the corresponding 19 Cα atoms from the three HAP2 structures discussed revealed an rmsd of <0.73 Å, highlighting the conservation of the structural core of the HAP2 domain II (Fig 2A and 2B). The conserved HAP2 salt bridge (E^126^–R^185^ in *Cr*HAP2 [1], E^117^–R^163^ in *At*HAP2, and E^121^–R^176^ in *Tc*HAP2) superposes such that the side chains fall on top of each other when the superposition is based on the *bdc* β-sheet Cα atoms (Fig 2B). Furthermore, alignment of the *bdc* β-sheets also brings into superposition the *ij* loop (which is included in the pfam10699 segment, conserved among identified HAP2 sequences, [1]) together with the α2 helix. The high structural conservation of the central core of domain II thus contrasts with the high variability of its membrane insertion region.

### An amphipathic α-helix is the membrane insertion element in HAP2 of flowering plants

**Fig 4:**
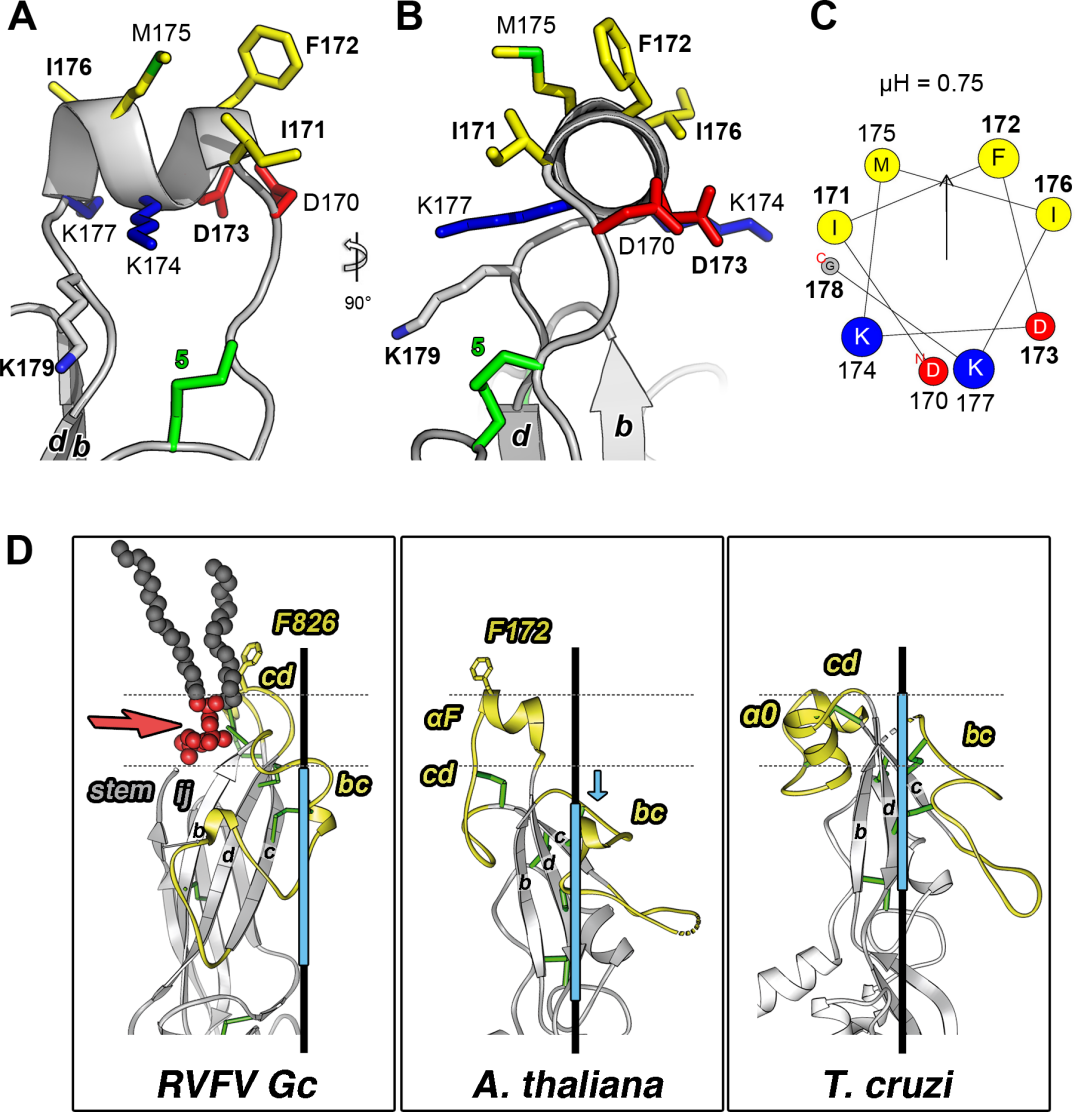
The *At*HAP2 fusion helix. (A-B) Gray ribbon representation of *At*HAP2 αF at the tip of domain II. Side chains of apolar (yellow), negatively charged (red), and positively charged (blue) residues are represented as sticks and labeled, with disulfides in green and numbered as in Fig 1; residues interrogated in subsequent experiments are in bold. (A) and (B) show two orthogonal views. (C) Helical wheel projection of αF; circle size is proportional to side chain size (amino acids colored as in A and B). The hydrophobic moment of the amphipathic α helix (μH) predicted by Heliquest [22] is displayed as a vertical arrow and its value indicated above the diagram. (D) The tip region of *At*HAP2 is displayed in the central panel, with *Tc*HAP2 on the right and the fusion protein Gc of the RVFV (PDB 6EGU; [13]) on the left, with a phosphatidyl-choline lipid bound in a glycerophospolipid-specific pocket in between the *cd* connection and the top end of the *bdc* β-sheet (the lipid is shown as connected spheres with the head group red and the aliphatic tails dark gray). The horizontal dotted lines mark roughly the borders of the polar lipid head group region of the membrane, based on this structure. In the central and right panel, the dotted lines show the predicted span of the polar head group region based on the aromatic residues that project into the aliphatic portion of the membrane. This location is clearer in the case of *At*HAP2, in which F172 is placed at the same level as F826 in the viral protein. In the case of *Tc*HAP2, the conformation of this region may have changed, as the nonpolar residues do not project out. The dotted lines are therefore only tentative. The trimer axis is shown in black, and the light blue bar indicates the region superposed in Fig 2B, which is shifted down in the case of *At*HAP2, as indicated by the blue arrow, such that the end of the *bdc* β-sheet is predicted to be away from the membrane. In all three panels, the *bc* and *cd* connections are in yellow, the conserved scaffold in gray, and disulfides as green sticks. *At*HAP2, *A*. *thaliana* HAP2; PDB, Protein Data Bank; RVFV, Rift Valley fever virus; *Tc*HAP2, *T*. *cruzi* HAP2

The *At*HAP2 fusion loop features an amphipathic helix (αF, Figs 2, 3 and 4) at its apex, positioned to interact parallel to the target membrane by insertion of its nonpolar surface (I^171^, F^172^, M^175^, I^176^) (Fig 4). Heliquest [22] predicted amphipathic helices in this segment for all plant and also for algal HAP2 sequences, including *Cr*HAP2 (S2 Fig, sequences in Fig 1B). This server also predicted an amphipathic helix in variable region 2 of the cnidarian animal *Nematostella vectensis* (S2 Fig), suggesting that this motif may be of widespread use for HAP2 target membrane insertion.

In contrast to *At*HAP2—in which the *bc* strand connection is basal to αF (Fig 2C) and away from the lipid contact area—in *Tc*HAP2, both *cd* and *bc* connections are located roughly at the same level at the tip of domain II, suggesting that, together, they may comprise the membrane insertion element (Fig 1C, Fig 4D, right panel). A surface representation of the modeled *Tc*HAP2 trimer indeed suggests a relatively flat membrane interaction surface with a tripartite nonpolar patch comprising V^129^ (in the N-terminal side of the *bc* connection) and L^167^, L^168^ and I^183^, F^184^ (in the variable regions 2 and 3, respectively, of the *cd* connection, S3A Fig). Compared to *Cr*HAP2, in *Tc*HAP2, the α0 helix features an additional helical turn after disulfide 5 (Fig 2B, compare left and middle panels). As anticipated from the aa sequence, the loop preceding α0 is stabilized by an extra disulfide bond, numbered 5b (Figs 1B and 2C, middle inset). The second loop (variable region 3) is just a turn connecting α0 to the *d* strand. Residues I^183^/F^184^ are located in the extra turn of α0 and are pointing inward (toward the *bdc* β-sheet) in the structure (S3 Fig), raising the possibility that the local conformation may be different in the aqueous solution used for crystallization than when bound to a lipid bilayer. A similar situation was observed for the fusion loops of the rubella virus fusion protein E1 [19].

The structural results, when combined with the sequence alignment, support the notion that HAP2 has evolved multiple modes for membrane insertion, with the algae having two fusion loops also predicted to bear amphipathic α-helices, only one loop in the flowering plants, and three small loops in the kinetoplastids (Figs 1B and 2C). The insertion mode of the RVFV Gc was shown to involve a lipid head group binding pocket located between the fusion loops and the end of the *bdc* β-sheet most proximal to the membrane (Fig 4D, left panel). In the case of *At*HAP2, the structure suggests a different insertion mode, as the *bdc* β-sheet is retracted from the membrane (blue arrow in Fig 4D) at a distance incompatible with interactions with lipid heads. In contrast, *At*HAP2 αF is poised to make multiple interactions within the hydrophilic region of the membrane (indicated roughly by the dotted lines in Fig 4D), while its nonpolar side can embed in the aliphatic moiety.

### The HAP2 ectodomains insert into liposomes as trimers via the domain II tip

**Fig 5:**
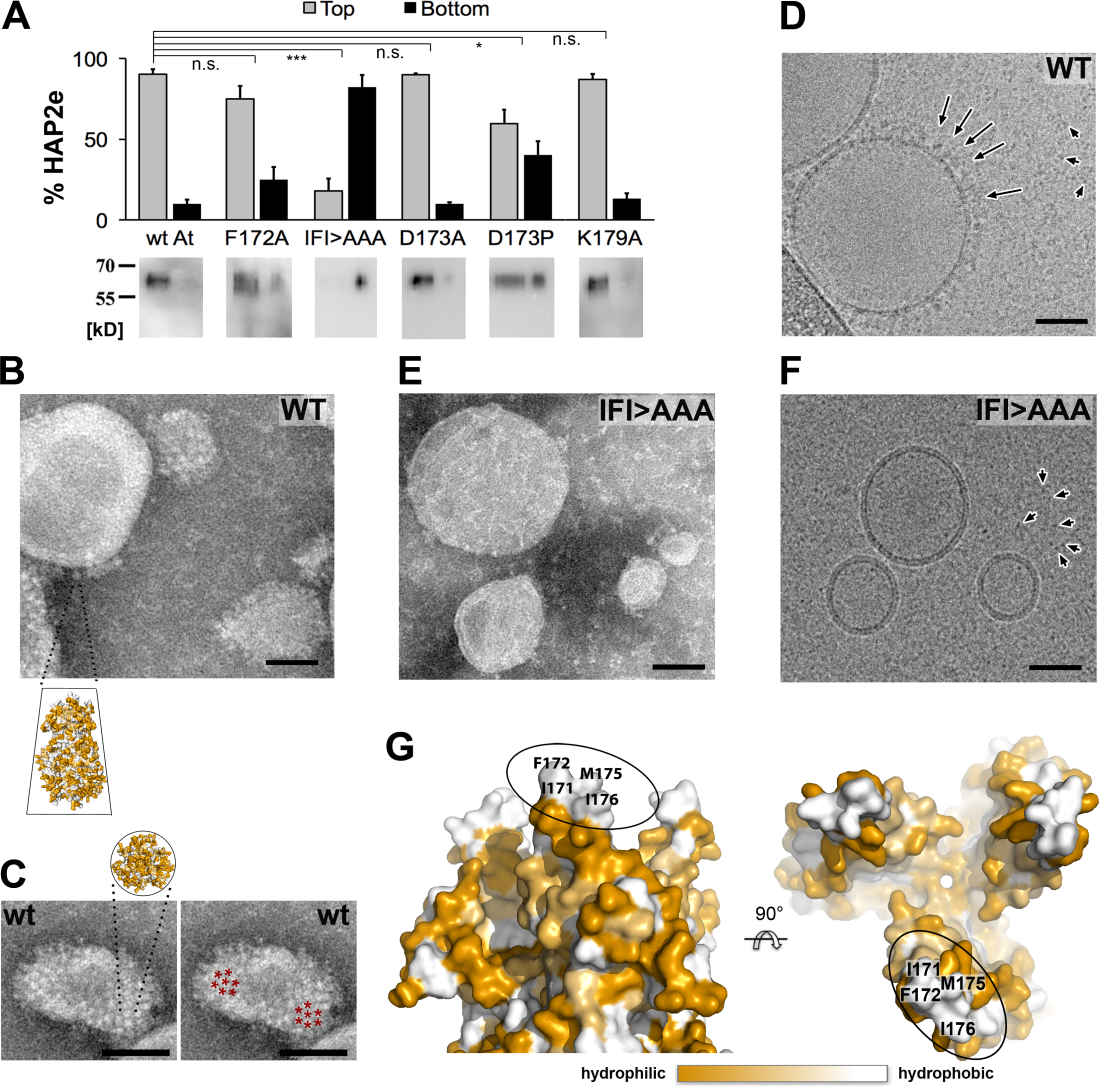
*At*HAP2e trimers insert into liposomes via the fusion helix. (A-F) Membrane insertion of *At*HAP2e. (A) *At*HAP2e membrane insertion was analyzed by differential sucrose gradient fractionation; the amount of HAP2e present in the top (gray) and bottom (black) fractions is shown as mean ± SD (see S1 Data); corresponding immunoblots are shown below. Statistical comparisons were performed using a Student *t* test. *p < 0.05; ***p < 0.001. Electron micrographs of liposomes incubated with WT HAP2e (B,C,D) and the IFI>AAA mutant (E,F). Samples were analyzed by negative stain EM (B,C,E) or cryo-EM (D,F). Scale bar 50 nm. Liposomes decorated with HAP2e display protein projections at the surface (B,C,D), contrasting with the smooth liposome surface in the presence of IFI>AAA mutant (E,F). The protein projections (some of which are indicated by long arrows in D) are rod shaped with a tapered membrane-proximal end—an inset illustrates the fitting of a trimeric *At*HAP2e into one projection (B). The background is coated with proteins not bound to liposomes (short arrows in D,F). (C) Top view of a liposome decorated with *At*HAP2e clustering in hexagonal arrays; an inset illustrates fitting of an *At*HAP2 trimer; red asterisks illustrate trimer clustering. (G) Surface representation of the membrane-facing tip of the *At*HAP2 trimer viewed from the side (left panel) and the top (right panel). Surface residues are colored according to hydrophilicity from dark orange (hydrophilic) to white (hydrophobic). The fusion loop region from one protomer is encircled, and hydrophobic residues that are exposed at the tip are labeled. *At*HAP2, *A*. *thaliana* HAPLESS 2; *At*HAP2e, *A*. *thaliana* HAPLESS 2 ectodomain; EM, electron microscopy; n.s., not significant; WT, wild type

To test that *At*HAP2 can indeed interact with membranes as predicted by the structures, we analyzed the behavior of *At*HAP2e by mixing the monomeric protein (S1B Fig) with liposomes of varying composition (see Materials and methods), followed by gradient ultracentrifugation. We designed the gradients so that proteins bound to liposomes would float to the top fractions, while the unbound protein would sediment. We found that the soluble, wild-type (WT) *At*HAP2e migrated to the top of the gradient, in contrast to a triple alanine substitution mutant in αF, which remained in the bottom of the tube (Fig 5A, S1 Data), indicating that HAP2 binds membranes and that the bulky nonpolar side chains of αF are required. Of note, we found that *At*HAP2 association with liposomes was enhanced when artificial membranes included the negatively charged phospholipid 1,2-dioleoyl-sn-glycero-3-phospho-L-serine (DOPS; see Materials and methods). Electron microscopy further showed that both *At*HAP2e (Fig 5B–5D) and *Tc*HAP2e (S3B and S3C Fig) decorated the liposome surface, as had been observed with *Cr*HAP2e. Side views of the proteoliposome edge showed approximately 12-nm-long projections with a tapered end toward the membrane (Fig 5B and 5D, S3A and S3B Fig), and top views showed a typical pattern of hexagonal packing of postfusion trimeric ectodomains at the liposome surface (Fig 5C), consistent with the overall shape, orientation, and lateral packing of postfusion trimers inserted into liposomes observed earlier for viral class II fusion proteins [19, 23, 24]. The size and shape of the projections observed on the HAP2e proteoliposomes are also compatible with a trimeric postfusion form and not with the monomers that were used to initiate the experiment (Fig 5B–5D), indicating that trimerization takes place upon interaction with lipids. Taken together, these results confirm that, as observed for *Cr*HAP2 and a number of viral class II fusion proteins, the interaction with membranes leads to trimerization and that the trimers interact with the membrane via the variable, nonpolar surface of the domain II tip. A similar trimerization process occurred in the crystallization drops in the case of *At*HAP2 and *Cr*HAP2, which were also set to crystallize as monomers. Trimerization in the crystallization drops was also described for the flavivirus class II protein, albeit at acidic pH [25]. In the HAP2 case, the trigger for trimerization—which is irreversible for class II fusion proteins [26]—is likely to have been the very high protein concentration used in the crystallization trials. The physiological trigger of HAP2 trimerization to induce gamete fusion in vivo remains to be understood.

### αF amphipathicity is required for *At*HAP2 in vitro liposome insertion and in vivo function

**Fig 6:**
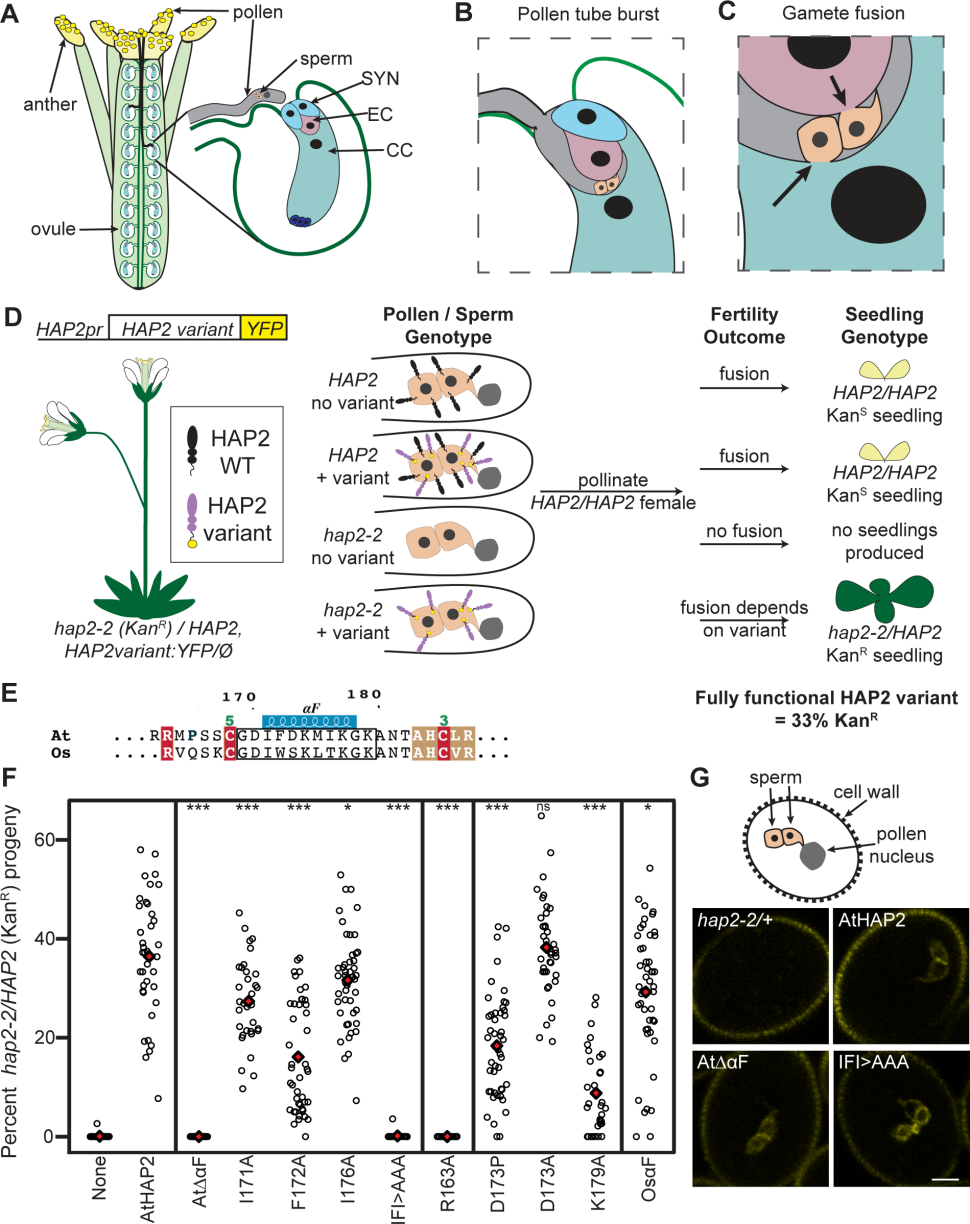
*Arabidopsis* gamete fusion requires presentation of hydrophobic residues within the amphipathic HAP2 αF helix. (A) Schematic of male gamete delivery and double gamete fusion within an *Arabidopsis* flower. Four anthers are shown surrounding the pistil, which contains two columns of ovules; petals and sepals are not depicted. Two pollen tubes (black lines) are shown targeting ovules. The inset illustrates a pollen tube approaching a female gametophyte, which develops within the ovule. A pair of sperm cells and the pollen tube nucleus are shown within the pollen tube; synergid cells (“SYN”), egg cell (“EC”), central cell (“CC”), and three antipodal cells (dark blue) are shown within the female gametophyte. (B) A schematic showing a pollen tube that has burst into a synergid, propelling the sperm to the site of fusion with the egg or central cell. (C) Arrows highlight the fusion of one sperm with the egg and the other with the central cell. (D) Assay for HAP2 function in vivo using transmission of *hap2-2* (T-DNA insertion carries kanamycin resistance, “Kan^R^”). *hap2-2/+* plants carrying WT or variant HAP2:YFP transgenes were produced. These plants will generate four pollen/sperm genotypes. Endogenous HAP2 (black) or introduced HAP2:YFP construct (purple/yellow) are shown at the sperm surface. The fertility outcome for each sperm genotype is indicated; a functional copy of HAP2 (either endogenous or a functional HAP2:YFP variant) must be expressed by sperm to produce a viable seed. Only *hap2-2* sperm that carry a functional HAP2:YFP variant transgene will sire Kan^R^ progeny when outcrossed to *ms1* females. Expression of a fully functional HAP2:YFP variant will result in 33% Kan^R^ seedlings. (E) The *Arabidopsis* (“*At*”) and rice (“*Os*”) HAP2 membrane interaction surfaces including the αF helix are shown; the region chosen for mutagenesis is identified with a box. (F) Quantitative analysis of effect of HAP2:YFP variants on *hap2-2* transmission (see S2 Data). Each data point (circle) represents the percentage of progeny from a single cross-pollination that inherited the *hap2-2* allele; the mean is indicated with a red diamond. A minimum of three independent transgenic lines were analyzed for each HAP2:YFP variant (see Materials and methods). HAP2:YFP variants tested are indicated on the x-axis: none, no transgene; others defined in text; *p*-values from the Wilcoxon rank sum test comparing each variant to *At*HAP2 are indicated: *p < 0.05, ***p < 0.001. (G) HAP2:YFP is detectable in sperm cells of transgenic plants. Confocal micrographs show HAP2:YFP signal from the indicated variant and autofluorescence of the pollen cell wall. Scale bar, 5 μm. A schematic depicts the arrangement of a pair of sperm cells within the pollen grain; the pollen nucleus and cell wall are depicted. *HAP2*, *HAPLESS 2*; *ms1*, *male sterile 1*; ns, not significant; T-DNA, transfer DNA; WT, wild type; YFP, yellow fluorescent protein

Flowering plant sperm are nonmotile and are delivered to female gametes in the cytoplasm of a pollen tube ([27], Fig 6A). Rupture of the pollen tube releases a pair of isogenic sperm cells; one fuses with the egg to produce a zygote, the other with the central cell to initiate endosperm development (Fig 6B and 6C). These two gamete fusion events are the defining feature of the flowering plants and are essential for the production of grain crops. To assess the role of αF in flowering plant gamete fusion, we utilized a genetic transmission assay (Fig 6D), which allowed us to determine whether HAP2 variants were able to restore gamete fusion to *Arabidopsis hap2-2* null mutant sperm. In crosses between WT (HAP2/HAP2) females and *hap2-2/HAP2* heterozygous males, the *hap2-2* allele is not inherited by progeny (Fig 6D, [28, 29]). However, if a transgene carrying fully functional HAP2 is introduced into *hap2-2/HAP2* plants, 33% of progeny will inherit *hap2-2* (Fig 6D, *At*HAP2 WT). This assay provided a quantitative readout for HAP2 function in vivo and facilitated dissection of the critical residues of the domain II tip (Fig 6D, S4 Table). We first tested whether *At*HAP2 αF is essential for function by deleting the helix along with two N- and C-terminal residues (box in Fig 6E, *At*ΔαF). Deletion of αF rendered HAP2 nonfunctional; *hap2-2* transmission rates were zero (Fig 6F, S2 Data, *At*ΔαF) even though expression of HAP2ΔαF:yellow fluorescent protein (YFP) in sperm was similar to the WT control (Fig 6G, S4 Fig).

We predicted that the nonpolar residues (I^171^, F^172^, M^175^, I^176^) on the nonpolar face of αF (Fig 4A–4C) would be critical for insertion into female gamete plasma membranes. To address this hypothesis, we tested whether these residues were important for association of *At*HAP2e with liposomes (Fig 5A). When we mutated F^172^ to alanine, membrane association was decreased, and when I^171^, F^172^, and I^176^ were mutated to alanine (IFI>AAA triple mutant), membrane association was almost completely abrogated; the majority of *At*HAP2e was found at the bottom of the sucrose gradient (Fig 5A) and was not associated with liposomes (Fig 5E and 5F). In our genetic analysis in vivo, mutating either I^171^ or I^176^ to alanine led to mild reductions in function (Fig 6F), while F^172^A more strongly reduced HAP2-induced gamete fusion (Fig 6F). The I^171^F^172^I^176^>AAA triple mutant completely eliminated HAP2 function in vivo (Fig 6F), in line with the abrogated association with liposomes observed in vitro (Fig 5A). We conclude that these hydrophobic αF residues, which are not individually essential for function, provide a hydrophobic surface at the domain II tip required for insertion into the egg and central cell plasma membrane to initiate HAP2-driven gamete fusion.

### The conserved E^117^–R^163^ salt bridge is essential for *Arabidopsis* gamete fusion

It was proposed that the salt bridge between the invariant R^185^ and E^126^ in *Cr*HAP2 (Figs 1B, 2B and 2C) constrains the domain II tip and helps present the predicted fusion loops to the target membrane [1]. However, *Tetrahymena thermophila (Tt)* mating was not eliminated when the corresponding arginine was mutated [5], raising the question of whether the proposed function is maintained across species. We found that *A*. *thaliana* gamete fusion was completely abolished upon mutation of the corresponding arginine residue to alanine (R^163^A, Fig 6F). The aa sequence of *Tt*HAP2 shows that there is a lysine two residues downstream from the conserved arginine (Fig 1B, 21st line, *Ciliophora* in the “Chromista” block). It is possible that given the plasticity of this region, a salt bridge between this lysine and the conserved glutamate in β-strand *b* can rescue the fusion activity, but further experiments with *Tt*HAP2 would be required to test this option.

### αF is interchangeable across the diversity of flowering plants

To determine whether the helical nature of αF is critical for *At*HAP2 function, we mutated D^173^—which is in the middle of the hydrophilic face of αF—to proline, a mutation predicted to introduce a kink in the helix. We found that D^173^P, but not D^173^A (which is expected to maintain helical character), reduced HAP2 function in vivo (Fig 6F) and reduced the ability of *At*HAP2e to associate with liposomes (Fig 5A), indicating that altering the helical conformation in this region affects membrane insertion. Both the length and amphipathicity of αF appear to be conserved among flowering plants (Fig 1B). In addition, Lysine 179 is invariant among plant HAP2 sequences (Fig 1B) and is situated just below αF (Fig 4A and 4B). We found that mutating K^179^ to alanine strongly reduced the function of HAP2 in the gamete fusion assay (Fig 6F) but did not affect the ability of HAP2e to insert into liposomes in vitro (Fig 5A). This result suggests that K^179^ is not required for membrane insertion but could be critical for a different stage of the gamete fusion reaction in plants.

To further address the sequence requirements for αF, we tested whether *At*αF could be functionally replaced with the corresponding amphipathic helix of rice (*Oryza sativa* [*Os*], S2 Fig), a distantly related flowering plant species. This replacement resulted in a chimeric HAP2 variant that was functional (*Os*αF, Fig 6E and 6F). *Os*αF maintains the amphipathic nature of the helix and shares an isoleucine at position 171 but substitutes a tryptophan at position 173 and a threonine at position 176 (S2 Fig). The interchangeability of αF suggests plasticity in the mode of interaction between sperm-expressed HAP2 and its target membranes, at least within the flowering plant lineage and in spite of around 150 million years of divergence [30]. In contrast, when the entire rice ectodomain was used to replace the *Arabidopsis* ectodomain, this chimeric protein was unable to restore function to *hap2* mutant sperm [29], indicating that other aspects of the gamete fusion mechanism (e.g., regulation of HAP2 activity by specific interaction with additional, lineage-specific proteins) have diverged within flowering plants.

## Discussion

### Variable modes of HAP2 membrane insertion across eukaryotes

In this study, we provide evidence at the primary sequence (Fig 1) and at the structural (Fig 2) levels that HAP2, while maintaining its overall structure (Fig 3), has evolved highly divergent membrane interaction motifs by way of focal diversification across the eukaryotes in which it was positively identified (S5 Fig). Analysis of the *HAP2* gene structure in intron-rich genomes (e.g., flowering plants, algae, protozoa, cnidarians, and insects) revealed the consistent presence of an intron between the regions encoding the domain II β-strands *b*, *c*, and *d* (S6 Fig), providing a potential route for focused diversification of the intervening functional loops via alteration of splicing patterns and/or exon shuffling during evolution. The X-ray structures of HAP2 from two representative organisms exhibiting a contrasting pattern of insertions and deletions in the loops at the domain II tip suggest very different modes of membrane insertion. In *Arabidopsis*, this motif consists of a single loop that projects the amphipathic αF helix for insertion into the lipid bilayer (Fig 4), while in *T*. *cruzi*, the membrane interaction surface comprises three small loops (Figs 2 and S3). Although the domain II tip of *Tc*HAP2 lacks an amphipathic helix and in this respect appears similar to viral class II proteins, such as that of RVFV Gc, the elements required for binding a glycerophospholipid head group in the latter (Figs 2B and 3D, [13]) are absent, suggesting that *Tc*HAP2 uses yet another way of interacting with the lipid head groups. Moreover, the amphipathic helix αF observed in the flowering plants (Fig 4) is unlike the membrane interaction surface described for any of the three structural classes of viral fusion proteins (Fig 2B, Fig 3). It will be interesting to determine the forces that have driven diversification of the HAP2 fusion loops. One possibility that needs to be explored is that female gametes have evolved specific plasma membrane compositions important for gamete fusion. We found that *At*HAP2 inserted more efficiently into artificial liposomes containing DOPS (see Materials and methods), a phosphatidylserine mimic. Understanding of female gamete lipid composition is currently limited to bulk membrane analysis in species with large and easily accessible oocytes [31, 32]. However, the use of genetically encoded phospholipid sensors is leading to increased awareness of important functions for even low-abundance anionic phospholipids (e.g., phosphatidylserine) and can now be used to define the membrane composition of small and inaccessible female gametes like those of the flowering plants [33].

### HAP2 membrane insertion via the fusion loops correlates with gamete fusion in vivo

We have shown that *Tc* and *At*HAP2e insert into artificial membranes (Fig 5, S3 Fig) and have identified key nonpolar residues on αF that are essential for *At*HAP2e membrane insertion in vitro (Fig 5A) and for gamete fusion in vivo (Fig 6). These data are consistent with previous genetic and biochemical analyses in *Chlamydomonas* [1] and with the recent finding that antibodies against the predicted fusion loops of *Plasmodium* HAP2 block parasitic gamete fusion and transmission [34]. The hypothesis that HAP2 functions by direct membrane insertion is also supported by experiments in which a synthetic peptide corresponding to the predicted fusion loop of *Tt*HAP2, associated with membranes [5].

Class II viral fusion proteins are activated by exposure to acidic pH upon entry into the host endosome and are positively and negatively regulated by partner viral proteins [26, 35]. Whether and how HAP2 is triggered and the nature of the potential partner proteins involved in negative or positive regulation of HAP2 function will be active areas of future research; it will be interesting to determine whether these mechanisms are lineage specific or broadly conserved. For example, in *Arabidopsis*, EGG CELL 1 (EC1) has been shown to activate HAP2 for fertilization only after the sperm cells are released from the pollen tube to female gametes [36].

Our results show a very strong correlation between the ability of *At*HAP2 to insert into membranes in vitro and its functionality in vivo, indicating that membrane insertion is an essential step in the fusion process, as demonstrated for the viral fusion proteins. Nevertheless, a recent report [37] proposed a HAP2 fusion mechanism similar to that of the *C*. *elegans* somatic fusion factor proteins (epithelial fusion failure 1 [EFF-1] and anchor-cell fusion failure 1 [AFF-1]; [38]), which do not function via target membrane insertion. These are the only members of the class II fusion protein structural family that lack a target membrane insertion surface at the domain II tip and were proposed to function instead by *trans*-oligomerization of proteins resident in the membranes of adjacent cells destined to fuse. Our findings do not support such a mechanism for HAP2, in line with the observation that in *Arabidopsis*, HAP2 is essential for sperm fertility but is not required for female fertility [28, 39]. A virus-like fusion mechanism is also in agreement with the observation that HAP2 is expressed in only one of the two gamete types to be fused in multiple plant, protozoan, and animal species in which it was studied [2, 3, 28, 39–41]. Indeed, expression from only one gamete is sufficient for gamete fusion in all species tested thus far [2, 28, 39, 40, 42], making the requirement for a second HAP2-like membrane fusion protein in the opposite gamete for fusion unlikely.

### Potential HAP2 signatures

HAP2 is the only gamete plasma membrane fusion protein to be identified thus far [43]. aa sequence analyses have identified a HAP2-specific motif (pfam10699, [44]) that has detected orthologs in four out of the five eukaryotic kingdoms ([1, 2, 4], Figs 1 and S5), suggesting that this protein was present in the last common ancestor to all eukaryotes and was a seminal innovation in the evolution of sexual reproduction. But these analyses have not identified orthologs in some well-studied clades like nematodes, vertebrates, and fungi (S5 Fig). While we cannot exclude the possibility that HAP2 was replaced in some lineages by a fusion protein of a different origin, its widespread but sporadic identification in eukaryotic genomes (S5 Fig) suggests the more likely scenario that for many organisms, its sequence has diverged enough to escape detection by traditional sequence-based searches. A possible evolutionary force that may have driven HAP2 divergence in some lineages is positive selection for sequence diversity to maintain barriers between species. Proteins mediating cellular interactions critical for fertilization are well known to diversify rapidly and reinforce interspecific fertility barriers [45]. Such interactions between HAP2 and partner proteins that regulate its fusion activity may have driven further divergence of the HAP2 sequence in organisms like yeast, *C*. *elegans*, mice, or humans, which currently lack candidate gamete fusion proteins.

The two HAP2 structures provided here (Fig 2A and Fig 3A), together with the previously reported *Cr*HAP2e [1], could be useful in defining a structural signature to identify additional orthologs. X-ray structures were required to identify viral class II fusion proteins because they lack any detectable sequence similarity across viral genera [26, 35]. A comparison of the structural conservation of the HAP2 domain II structure across eukaryotes (Fig 3A) with that of the class II fusion proteins from different viral families (Fig 3B) shows that diverse HAP2 molecules conserve the relative orientation and length of most of its secondary structure elements, while the viral class II proteins show higher variation. Indeed, the HAP2 structures share an α2 helix of identical length and orientation, the same organization of the *ij* loop, and the core *bdc* β-sheet, including an invariant salt bridge anchoring the variable fusion loops to the central core of the molecule. In comparison, the viral class II proteins (Fig 3B), in spite of sharing the same elements within domain II (α2 helix, *ij* hairpin, core *bdc* β-sheet), differ in their relative orientations and positioning and are thus more structurally diverse. This is clearly seen in the table of DALI scores (S3 Table), which reveals a large gap in between the very similar HAP2 domain II structures and the more divergent domain II structures from the other known class II proteins. This gap suggests the possibility that HAP2 orthologs with much lower sequence identity may be found that display the same three-dimensional fold. HAP2 orthologs identified thus far share approximately 30% sequence identity between the aligned residues across domain II, whereas viral fusion proteins are only about 10% identical in the aligned residues. It is thus plausible that additional HAP2 orthologs with lower sequence identity exist at a level insufficient for sequence-based identification. Irrespective of whether class II fusion proteins have a viral or cellular origin [46], the fact that the viral evolutionary clock is several orders of magnitude faster than its cellular counterpart suggests that HAP2 may have maintained an ancestral organization relative to the class II proteins of present-day viruses. The high conservation of the HAP2 core structure further suggests that bioinformatics approaches should be able to translate the observed structural similarity into signatures detectable in more distant HAP2 sequences, for instance, by analyzing the covariance of interacting residues distant in the sequence.

## Materials and methods

### Plant growth conditions

Seeds were stratified (at least 2 days at 4°C) on solid Murashige and Skoog (MS) medium (Sigma Aldrich, St. Louis, MO) supplemented with appropriate antibiotics and germinated at 22°C under constant light (Percival incubator). After 7–14 days, seedlings were transferred to sterile #2MIX potting media (www.fafard.com) with fertilizer (N:P:K, 15:5:15) and were grown at 20°C, 50%–60% humidity, on a 16-hour-light / 8-hour-dark light cycle in growth chambers (Environmental growth chambers, Chagrin Falls, OH, United States of America).

### S2 cell lines

*Drosophila* S2 cells (ATCC CRL-1963) were cultured in Schneider’s complete media (Thermo Scientific) before transfection and in Insect Xpress media after transfection (Lonza, Basel, Switzerland). Culturing and transfection of S2 cells has been described previously [47].

### Expression and purification of the soluble HAP2 ectodomain

Codon-optimized synthetic cDNA corresponding to a soluble C-terminally truncated version of the HAP2e comprising residues 25–494 from *A*. *thaliana* and 26–516 from *T*. *cruzi* were cloned into a modified *Drosophila* S2 expression vector described previously [48], and transfection was performed as reported earlier [47]. For large-scale productions, cells were induced with 4 μM CdCl_2_ at a density of approximately 7 × 10^6^ cells/ml for 8 days and pelleted, and the soluble ectodomains were purified by affinity chromatography from the supernatant using a StrepTactin Superflow column followed by SEC using a Superdex200 column in 10 mM TRIS pH8 100 mM NaCl. Pure proteins were concentrated to approximately 8 and 4 mg/ml, respectively.

### SEC with MALS

Purified HAP2e were subjected to SEC using a Superdex 200 column (GE HealthCare) equilibrated with 10 mM TRIS pH8 100 mM NaCl. Separation was performed at 20°C with a flow rate of 0.5 ml min^−1^. Online MALS detection was performed with a DAWN-HELEOS II detector (Wyatt Technology, Santa Barbara, CA, USA) using a laser emitting at 690 nm. Online differential refractive index measurement was performed with an Optilab T-rEX detector (Wyatt Technology). Data were analyzed, and weight-averaged molecular masses (Mw) and mass distributions (polydispersity) for each sample were calculated using the ASTRA software (Wyatt Technology).

### Liposome coflotation experiments

DOPS, 1,2-dioleoyl-sn-glycero-3-phosphoethanolamine (DOPE), 1,2-dioleoyl-sn-glycero-3-phosphocholine (DOPC), cholesterol, and sphingomyelin were purchased from Avanti Polar Lipids. Liposomes were freshly prepared in PBS by the freeze-thaw and extrusion method using molar ratios of 17% DOPE, 16% DOPC, 50% cholesterol, 17% sphingomyelin. Alternatively, for coflotation assays on WT and mutant *At*HAP2e proteins, the following alternative composition was used: 50% cholesterol, 30% DOPC, 20% DOPS. Then, 0.07 μM purified HAP2e was mixed with 8 mM liposomes and incubated for 1 hour at 21°C in 100 μL PBS. Samples were then adjusted to a final concentration of 20% sucrose, overlaid with a 5%–60% sucrose gradient (in PBS), and centrifuged for 1 hour at 4°C at approximately 150,000 × g. Fractions from the top, middle, and bottom of the gradient were analyzed by immunoblotting using a monoclonal anti-strep tag antibody, and the bands quantified using the GeneTools Syngene software. The percentage of HAP2e in either fraction was calculated as the ratio between HAP2e in individual fractions and total HAP2e (sum of HAP2e in top and bottom fractions).

### Electron microscopy

Purified HAP2e (*A*. *thaliana* or *T*. *cruzi*) mixed with liposomes was spotted on glow-discharged carbon grids (CF300, EMS, USA), negatively stained with 2% phosphotungstic acid (PTA) PH 7.4, analyzed with a Tecnai G2 Bio-Twin electron microscope (FEI, USA), and imaged with an Eagle camera (FEI, USA). For cryo-electron microscopy, liposomes mixed with purified HAP2e were applied on a glow-discharged Lacey Carbon grid (Agar Scientific, UK). Samples were plunge-frozen in liquid ethane using an automated system (Leica EMGP, Austria) and visualized on a Tecnai F20 electron microscope operating at a voltage of 200 kV. Image frames were recorded in low-dose mode on a Falcon II direct electron detector (FEI, USA).

### Crystallization and structure determination

Crystals of HAP2e from *A*. *thaliana* were grown at 293 K using the hanging-drop vapor-diffusion method in drops containing 1 μL protein solution mixed with 1 μL reservoir solution containing 100 mM Sodium Citrate pH 4.5 and 200 mM zinc acetate. Diffraction-quality rod-like crystals appeared after 1 week and were flash-frozen in mother liquor containing 30% (v/v) glycerol. The crystals diffracted anisotropically to 2.24 Å along the c* axis but only to 3.7 Å in orthogonal directions. Therefore, an ellipsoidal cut off was applied using the StarAniso server (STARANISO version 1.7.2 18-Apr-17 Ian J. Tickle, Global Phasing, Cambridge, UK; http://staraniso.globalphasing.org/cgi-bin/staraniso.cgi) in order to remove noise, and refinement was carried out to a nominal resolution of 2.75 Å.

Limited proteolysis of HAP2e from *T*. *cruzi* was carried out by adding subtilisin (dissolved in 10 mM Tris pH8, 30 mM NaCl at 10 mg/mL) to a solution containing *T*. *cruzi* HAP2e at 16 mg/mL in 10 mM Tris pH8, 100 mM NaCl at a 1:300 w:w ratio. After 1 hour of incubation at room temperature, the protease was inactivated by addition of 1 mM PMSF, and the protease-resistant fragment accounting to approximately 60% of the digested protein was purified by SEC using a Superdex200 column (*Tc*HAP2e_sub_). Crystals of *Tc*HAP2e_sub_ were grown at 293 K using the hanging-drop vapor-diffusion method in drops containing 1 μL protein solution mixed with 1 μL reservoir solution containing 100 mM CHES pH 9.0, 200 mM NaCl, and 10% w/v PEG 8k. Diffraction-quality rodlike crystals appeared after 7–10 days and were flash-frozen in mother liquor containing 25% (v/v) glycerol.

Data collection was carried out at the ESRF (ID30A-3) and the Synchrotron Soleil (Proxima-1). Data were processed, scaled, and reduced with XDS [49], Pointless [50], and programs from the CCP4 suite [51]. For the structure of *A*. *thaliana* HAP2e, the full *C*. *reinhardtii* HAP2 monomer (PDB 5MF1) was used to create a search model with Sculptor [52]. The so-called tip region was deleted (*bc* and *cd* connections). The structure was determined by the molecular replacement method using Phaser [53]. Zinc atoms were localized in the density by calculating an anomalous difference map using ANODE [54]. To determine the structure of the protease-resistant fragment of *T*. *cruzi* HAP2, we initially used Sculptor [52] to create a search model for molecular replacement based on the structure of a protomer of the *C*. *reinhardtii* HAP2 trimer (PDB 5MF1). The full monomer model was divided into 3 individual domains (domains I, II, and III), and loops and exposed side chains were trimmed off. The structure was finally determined by the molecular replacement method using Phaser [53] and an isolated domain II as search model. Phases were refined using the anomalous signal of a highly redundant Sulfur-SAD data set collected at a wavelength of 2.06641 A on crystals of the native protein.

For both orthologs, model building was performed using Coot [55], and refinement was done using AutoBuster [56].

### Generation of a trimeric model of *Tc*HAP2

Two independent *Tc*HAP2 trimer models were generated from superposition on either the *At*HAP2 or *Cr*HAP2 using the secondary structure matching function in Coot [55], and the resulting two *Tc*HAP2 trimers were structurally very similar, with rmsd = 2.6 Å for 711 common Cα atoms (237 per subunit).

### Generating *hap2-2/HAP2* transgenic plants carrying HAP2:YFP variants

*Arabidopsis HAP2* coding sequence variants were generated using a transfer-DNA plasmid containing the native *HAP2* promoter and coding sequence with a C-terminal YFP fusion (*pHAP2*:*HAP2cds*:*YFP*; PGL290; CDS, GenBank AAY51999.1; pGreen [57] backbone; Basta-resistant seedlings). Mutations were made using the NEB Q5 Site-Directed Mutagenesis Kit (NEB #E0554S); primer sequences are provided in S5 Table. Mutations were confirmed by sequencing (Genewiz, South Plainfield, NJ). *Agrobacteria tumefaciens*–mediated transformation (strain GV3101 [58] with pSoup helper plasmid [57]) of *hap2-2/HAP2* (SALK_152706; [28, 59]) was performed by floral dip [60]. *hap2-2/HAP2* seedlings were selected on MS plates supplemented with sucrose (5 g/L) and kanamycin sulfate (50 mg/L; Life Technologies/Thermo Fisher Scientific, Waltham, MA).

After seeds were collected from transformed plants, they were sterilized using a 50% bleach solution containing 0.02% Triton X-100 for 7 minutes, followed by washing 4 times with sterilized water. Seeds were resuspended in sterilized 0.1% agarose and were plated on 15-cm dishes containing MS media supplemented with Basta (25 mg/L; Chem Service, West Chester, PA; Oakwood Chemical, Estill, SC). After at least 2 days at 4°C, plates were moved to a 22°C incubator with constant light. After 7–14 days, Basta-resistant seedlings were transferred to soil made up with 1X fertilizer (4 plants / pot). For primary transformants (T_1_), DNA was isolated using the leaf boil method [61]. T_1_ plants were genotyped for the *hap2-2* allele using primer sets to detect both the Salk tDNA insertion (LbaI, [59]; hap2seqTR3 [28]) and WT genomic HAP2 (hap2c2; hap2seqTR3; see S5 Table).

### Genetic transmission assay for assessing transmission of *hap2-2* allele

Pollen from T_1_ plants that were heterozygous for *hap2-2/+* were hand-pollinated onto *male sterile 1 (ms1-1)* pistils [62]. Seeds from individual *ms1* crosses were gas-sterilized and plated on MS Kanamycin (50 mg/L) plates supplemented with sucrose (5 g/L). Seeds were cold-treated at 4°C for at least 2 days and then transferred to a 22-°C incubator with constant light. *hap2-2/+* seeds were simultaneously plated on MS Kanamycin plates to ensure the drug resistance was working correctly. For analysis of *hap2-2* transmission, resistant versus sensitive seedlings were counted manually after 6–10 days at 22°C.

Mutations were confirmed in at least one T_1_ line per transgene by sequencing of a PCR product. PCR products were generated using a EcoRI-HAP2-CVFP-F1 forward primer and the HAP2Ex7R reverse primer. For HAP2 variants that had 0% transmission of *hap2-2*, seeds were collected from the T1 plant and plated on MS Kanamycin and MS Basta separately to confirm segregation of the *hap2-2* allele and the transgene, respectively, in the next generation.

### Analysis of HAP2:YFP variant expression

Expression of HAP2:YFP in *pHAP2*:*HAP2variant*:*YFP* transgenic plants was initially screened using fluorescence microscopy. Pollen from T_1_ plants was hydrated on slides in pollen growth media [63] and imaged using a Zeiss Axiovert 200M microscope (images not shown). Each transgenic line was analyzed using confocal microscopy (Fig 5G, S4 Fig) of the T_1_ and/or T_2_ generation. Pollen were hydrated in pollen growth media and imaged using a Zeiss LSM 800 confocal microscope. All images were taken using excitation with the 488 laser at 10% (488 settings kept constant) and using the 40× water objective with a 2× zoom. Single optical slices were collected (974 × 974 pixels, 16-bit). All images were imported to ImageJ (FIJI version) [64] and cropped to 300 × 300 pixels.

### Statistical analysis

Data are presented as mean ± SD unless otherwise indicated in figure legends, and experimental repeats are indicated in figure legends.

The following criteria were used to analyze HAP2:YFP variant transgenic plants: at least three independent transgenic lines were analyzed, at least three crosses were performed for each line, and only crosses with at least 10 seeds were analyzed (S4 Table). Transmission data did not follow a normal distribution for each transgene (confirmed by Anderson-Darling test for normality in R studio [65], using R version 3.2.3 [66]); therefore, a nonparametric statistical test was used. Each HAP2 mutant was compared to the WT transgene (*At*HAP2) using a Wilcoxon rank sum test with continuity correction in R studio.

### Data deposition

The atomic coordinates and structure factors for two structures have been deposited in the PDB under the accession numbers 5OW3 (*At*HAP2) and 5OW4 (*Tc*HAP2).

## Supporting information

S1 Fig Biochemical characterization of *At*HAP2e and *Tc*HAP2e and crystal analysis. (A) *At*HAP2e and (C) *Tc*HAP2e purity was assessed by Coomassie blue–stained SDS-PAGE. SEC analysis of *At*HAP2e (C) and (D) *Tc*HAP2e on a Superdex 200 column in 10 mM Tris pH8, 100 mM NaCl revealed the presence of a single peak. SEC-MALS analysis indicated that this peak for both *Tc* and *At*HAP2e corresponds to a monomer. The y-axis on the right gives the UV absorption at 280 nm on a relative scale from 0 to 1 (plotted in black), while the y-axis on the left gives the molecular mass by MALS (plotted and labeled in red). (E) *Tc*HAP2e and the purified protease-resistant fragment resulting from subtilisin cleavage (*Tc*HAP2e_sub_) were separated by SDS-PAGE stained with Coomassie blue. (F+G) Overview (top panel) and close-up (bottom panel) of the *Tc*HAP2e_sub_ crystal packing. Domain II (yellow) assembles as a rigid dodecameric ring, whereas domain I projects into solvent within this ring, explaining why domain I is only partially traceable in the experimental electron density. (H) Composite omit electron density of part of the *cd* strand connection of *Tc*HAP2 (left) and *At*HAP2 (right) corresponding to variable regions 2 and 3, respectively, contoured at 0.9σ, allowing for manual building of the model in this region. *At*HAP2e, *A*. *thaliana* HAPLESS 2 ectodomain; MALS, multiangle static light scattering; SEC, size exclusion chromatography; *Tc*HAP2e, *T*. *cruzi* HAPLESS 2 ectodomain.
(TIF)

S2 Fig Amphipathic helix prediction in the variable region 3 in a subset of HAP2 orthologs. Helical wheel projections of HAP2 loop 2 residues from indicated organisms. The circle size is proportional to the size of the displayed side chain. Nonpolar residues are colored in yellow. Charged residues are colored red (negative charge) and blue (positive charge). Polar uncharged residues are purple and light pink. Glycines are displayed in gray, prolines in green. The hydrophobic moment value of the respective amphipathic helix predicted by Heliquest (μH) is shown below the organism name, suggesting the formation of an amphipathic helix in the putative membrane-interacting region of plant and algal HAP2 but also in some animal HAP2 orthologs. HAP2, HAPLESS 2.
(TIF)

S3 Fig *Tc*HAP2e trimers insert into liposomes via the hydrophobic domain II tip. (A) Surface representation of the membrane-facing tip of the putative *Tc*HAP2 trimer, obtained as described in Materials and methods, viewed from the membrane (left panel) and from the side (right panel). Surface residues are colored according to hydrophilicity from dark orange (hydrophilic) to white (hydrophobic). The fusion loop region from one protomer is encircled, and hydrophobic residues that are exposed at the tip are labeled. Residue 129 belongs to the *bc* connection, the others to *cd*. (B-C) Membrane insertion of *Tc*HAP2e. Electron micrographs of liposomes incubated in presence of HAP2e were analyzed by negative staining (B) or cryo-EM (C). Scale bar 50 nm. Liposomes decorated with HAP2e display protein projections at the surface. The protein projections (some of which are indicated by long arrows in C) are shaped as tapered rods similar to the ones of *At*HAP2e (see Fig 4) and form lateral assemblies. The background is coated with proteins not bound to liposomes (short arrows in C). *At*HAP2e, *A*. *thaliana* HAPLESS 2 ectodomain; EM, electron microscopy; *Tc*HAP2, *T*. *cruzi* HAPLESS 2; *Tc*HAP2e, *T*. *cruzi* HAPLESS 2 ectodomain.
(TIF)

S4 Fig Analysis of HAP2variant:YFP accumulation in sperm cells. Confocal micrographs of pollen grains from three independent transgenic lines expressing the indicated HAP2:YFP variant construct are shown. Each pollen grain shown contains a pair of sperm representing the maximum YFP intensity observed in the represented transgenic line. Signal from the pollen grain wall is due to autofluorescence and observed in the *hap2-2/+* control, which does not contain a HAP2:YFP construct. S4 Table lists the number of independent transgenic lines that were tested for each HAP2:YFP variant using our genetic transmission assay for HAP2 function (Fig 6). The average percent transmission of *hap2-2* (33% is expected for a fully functional single-insertion-site line) for each line is indicated in the bottom-right corner of each image. Scale bar, 5 μm. HAP2, HAPLESS 2; YFP, yellow fluorescent protein.
(TIF)

S5 Fig Scattered HAP2 positive identification across eukaryotic taxa. A schematic phylogeny representing the five eukaryotic kingdoms, subkingdoms, and phyla for which whole-genome sequence information is available (classification system; [17, 67]). Selected groups of species are provided for some phyla. Red font indicates presence of pfam10699 (the HAP2-GCS1 domain). Filled red circles: all available genomes of the phylum contain pfam10699; open red circles: the phylum contains examples both of genomes that have or do not have pfam10699; filled black circles: phyla in which none of the available genomes contains pfam10699. HAP2 was identified in *D*. *melanogaster* and *Apis mellifera* [4], but these sequences (and genomes) lack pfam10699 (blue font). Parenthetical numbers indicate the total number phyla within the subkingdom. Only phyla with at least one whole-genome sequence with >10,000 annotated proteins are listed. Bracketed numbers indicate the number of species from each phylum used in this analysis. GCS1, GENERARATIVE CELL SPECIFIC 1; HAP2, HAPLESS 2.
(TIF)

S6 Fig The region of HAP2 encoding β-strands *b*, *c*, and *d* frequently contains introns. The HAP2 gene organization from organisms representing four eukaryotic kingdoms (Plantae: *A*. *thaliana*, *C*. *reinhardtii*; Chromista: *Plasmodium falciparum*, *T*. *thermophila*; Protozoa: *T*. *cruzi*, *Dictyostelium discoideum*; Animalia, *N*. *vectensis*, *T*. *castaneum*, *Acyrthosiphon pisum*; S1 Table). Sequences have been ordered by decreasing organismal intron density (introns/1-kb coding sequence [68], in parentheses next to species name). The *T*. *cruzi* genome contains only a few introns, so intron density was not calculated (“nc”, [68]). Segments encoding the *bdc* β-sheet are highlighted (yellow) together with the location of the pfam10699 segment (purple) and the TM segments (green). TMs were identified from Uniprot for *C*. *reinhardtii* (A4GCR6.2), *A*. *thaliana* (F4JP36.1), and *T*. *thermophila* (A0A060A682) or by prediction (TMHMM server [69] or the Split 4.0 server [70]). TM predictions for *N*. *vectensis* were previously published [71]. Note that the β-strands *b*, *c*, and *d* are only encoded by one single exon in organisms with very few introns, suggesting a potential mode of evolution via focal variation at the interstrand connections. HAP2, HAPLESS 2; TM, transmembrane.
(TIF)

S1 Table Accession numbers of amino acid sequences used in this study. 1. (PDF)

S2 Table Data collection, phasing, and refinement statistics. (PDF)

S3 Table Structural similarity across class II fusion proteins. (PDF)

S4 Table Transgenic lines used to analyze function of HAP2:YFP variants. HAP2, HAPLESS 2; YFP, yellow fluorescent protein.
(PDF)

S5 Table Primers used for generation of HAP2:YFP variants and for genotyping and sequencing transformed plants. HAP2, HAPLESS 2; YFP, yellow fluorescent protein.
(PDF)

S1 Data Quantification of membrane insertion analysis for AtHAP2e and mutants. (Abbreviations: AtHAP2e, Arabidopsis thaliana HAPLESS 2 ectodomain).
(XLSX)

S2 Data Seedling counts used to assess in vivo function of HAP2:YFP variants. Seedling counts are provided as kanamycin sensitive (KanS) and kanamycin resistant (KanR). Total sensitive and resistant seedlings are listed (Total(R+S)) as well as percent transmission of kanamycin resistance (%KanR). HAP2:YFP variant (Variant) and transgenic line (Transgene_line) are indicated. (Abbreviations: HAP2, HAPLESS 2; YFP, yellow fluorescent protein).
(XLSX)

## Abbreviations

aa: amino acid
AFF-1: anchor-cell fusion failure 1
*At*HAP2e: *Arabidopsis thaliana* HAPLESS 2 ectodomain
*Cr*HAP2e: *Chlamydomonas reinhardtii* HAPLESS 2 ectodomain
DOPC: 1,2-dioleoyl-sn-glycero-3-phosphocholine
DOPE: 1,2-dioleoyl-sn-glycero-3-phosphoethanolamine
DOPS: 1,2-dioleoyl-sn-glycero-3-phospho-L-serine
EC1: EGG CELL 1
EFF-1: epithelial fusion failure 1
HAP2: HAPLESS 2
MALS: multiangle static light scattering
MS: Murashige and Skoog
ms1: *male sterile 1*
Os: *Oryza sativa*
PDB: Protein Data Bank
PTA: phosphotungstic acid
rmsd: root-mean-square deviation
RVFV: Rift Valley fever virus
S2: Schneider 2
SEC: Size exclusion chromatography
SFV: Semliki Forest virus
T_1_: primary transformant
TBEV: tick-borne encephalitis virus
*Tc*HAP2e: *Trypanosoma cruzi* HAP2 ectodomain
Tt: *Tetrahymena thermophila*
WT: wild type
YFP: yellow fluorescent protein

## References

[1] Fedry J, Liu Y, Pehau-Arnaudet G, Pei J, Li W, Tortorici MA, et al The Ancient Gamete Fusogen HAP2 Is a Eukaryotic Class II Fusion Protein. Cell. 2017;168(5):904–15 e10. 10.1016/j.cell.2017.01.024 ; PubMed Central PMCID: PMCPMC5332557. 28235200 PMC5332557

[2] Liu Y, Tewari R, Ning J, Blagborough AM, Garbom S, Pei J, et al The conserved plant sterility gene HAP2 functions after attachment of fusogenic membranes in Chlamydomonas and Plasmodium gametes. Genes & development. 2008;22(8):1051–68. 10.1101/gad.1656508 . 18367645 PMC2335326

[3] Steele RE, Dana CE. Evolutionary history of the HAP2/GCS1 gene and sexual reproduction in metazoans. PLoS ONE. 2009;4(11):e7680 10.1371/journal.pone.0007680 . 19888453 PMC2766252

[4] Liu Y, Pei J, Grishin N, Snell WJ. The cytoplasmic domain of the gamete membrane fusion protein HAP2 targets the protein to the fusion site in Chlamydomonas and regulates the fusion reaction. Development. 2015;142(5):962–71. Epub 2015/02/07. 10.1242/dev.118844 ; PubMed Central PMCID: PMCPMC4352976. 25655701 PMC4352976

[5] Pinello JF, Lai AL, Millet JK, Cassidy-Hanley D, Freed JH, Clark TG. Structure-function studies link class II viral fusogens and the ancestral gamete fusion protein HAP2. Current biology: CB. 2017. In press. 10.1016/j.cub.2017.01.049 PMC5393271 28238660

[6] Harrison SC. Viral membrane fusion. Nat Struct Mol Biol. 2008;15(7):690–8. 10.1038/nsmb.1456 ; PubMed Central PMCID: PMCPMC2517140. 18596815 PMC2517140

[7] Dessau M, Modis Y. Crystal structure of glycoprotein C from Rift Valley fever virus. Proc Natl Acad Sci U S A. 2013;110(5):1696–701. 10.1073/pnas.1217780110 ; PubMed Central PMCID: PMCPMC3562824. 23319635 PMC3562824

[8] Lescar J, Roussel A, Wien MW, Navaza J, Fuller SD, Wengler G, et al The Fusion glycoprotein shell of Semliki Forest virus: an icosahedral assembly primed for fusogenic activation at endosomal pH. Cell. 2001;105(1):137–48. . 11301009 10.1016/s0092-8674(01)00303-8

[9] Rey FA, Heinz FX, Mandl C, Kunz C, Harrison SC. The envelope glycoprotein from tick-borne encephalitis virus at 2 A resolution. Nature. 1995;375(6529):291–8. 10.1038/375291a0 . 7753193

[10] Harrison SC. Viral membrane fusion. Virology. 2015;479–480:498–507. Epub 2015/04/14. 10.1016/j.virol.2015.03.043 ; PubMed Central PMCID: PMCPMC4424100. 25866377 PMC4424100

[11] Apellaniz B, Huarte N, Largo E, Nieva JL. The three lives of viral fusion peptides. Chem Phys Lipids. 2014;181:40–55. Epub 2014/04/08. 10.1016/j.chemphyslip.2014.03.003 ; PubMed Central PMCID: PMCPMC4061400. 24704587 PMC4061400

[12] Lorieau JL, Louis JM, Bax A. The complete influenza hemagglutinin fusion domain adopts a tight helical hairpin arrangement at the lipid:water interface. Proc Natl Acad Sci U S A. 2010;107(25):11341–6. 10.1073/pnas.1006142107 ; PubMed Central PMCID: PMCPMC2895095. 20534508 PMC2895095

[13] Guardado-Calvo P, Atkovska K, Jeffers SA, Grau N, Backovic M, Perez-Vargas J, et al A glycerophospholipid-specific pocket in the RVFV class II fusion protein drives target membrane insertion. Science. 2017;358(6363):663–7. 10.1126/science.aal2712 . 29097548

[14] Guardado-Calvo P, Bignon EA, Stettner E, Jeffers SA, Perez-Vargas J, Pehau-Arnaudet G, et al Mechanistic Insight into Bunyavirus-Induced Membrane Fusion from Structure-Function Analyses of the Hantavirus Envelope Glycoprotein Gc. PLoS Pathog. 2016;12(10):e1005813 10.1371/journal.ppat.1005813 ; PubMed Central PMCID: PMC5082683. 27783711 PMC5082683

[15] Halldorsson S, Behrens AJ, Harlos K, Huiskonen JT, Elliott RM, Crispin M, et al Structure of a phleboviral envelope glycoprotein reveals a consolidated model of membrane fusion. Proc Natl Acad Sci U S A. 2016;113(26):7154–9. 10.1073/pnas.1603827113 . 27325770 PMC4932967

[16] Edgar RC. MUSCLE: a multiple sequence alignment method with reduced time and space complexity. BMC Bioinformatics. 2004;5:113 10.1186/1471-2105-5-113 ; PubMed Central PMCID: PMCPMC517706. 15318951 PMC517706

[17] Ruggiero MA, Gordon DP, Orrell TM, Bailly N, Bourgoin T, Brusca RC, et al A higher level classification of all living organisms. PLoS ONE. 2015;10(4):e0119248 10.1371/journal.pone.0119248 ; PubMed Central PMCID: PMC4418965. 25923521 PMC4418965

[18] Gibbons DL, Vaney MC, Roussel A, Vigouroux A, Reilly B, Lepault J, et al Conformational change and protein-protein interactions of the fusion protein of Semliki Forest virus. Nature. 2004;427(6972):320–5. 10.1038/nature02239 . 14737160

[19] DuBois RM, Vaney MC, Tortorici MA, Kurdi RA, Barba-Spaeth G, Krey T, et al Functional and evolutionary insight from the crystal structure of rubella virus protein E1. Nature. 2013;493(7433):552–6. 10.1038/nature11741 . 23292515

[20] Bressanelli S, Stiasny K, Allison SL, Stura EA, Duquerroy S, Lescar J, et al Structure of a flavivirus envelope glycoprotein in its low-pH-induced membrane fusion conformation. The EMBO journal. 2004;23(4):728–38. 10.1038/sj.emboj.7600064 . 14963486 PMC380989

[21] Holm L, Park J. DaliLite workbench for protein structure comparison. Bioinformatics. 2000;16(6):566–7. Epub 2000/09/12. . 10980157 10.1093/bioinformatics/16.6.566

[22] Gautier R, Douguet D, Antonny B, Drin G. HELIQUEST: a web server to screen sequences with specific alpha-helical properties. Bioinformatics. 2008;24(18):2101–2. 10.1093/bioinformatics/btn392 . 18662927

[23] Gibbons DL, Erk I, Reilly B, Navaza J, Kielian M, Rey FA, et al Visualization of the target-membrane-inserted fusion protein of Semliki Forest virus by combined electron microscopy and crystallography. Cell. 2003;114(5):573–83. . 13678581 10.1016/s0092-8674(03)00683-4

[24] Stiasny K, Allison SL, Schalich J, Heinz FX. Membrane interactions of the tick-borne encephalitis virus fusion protein E at low pH. J Virol. 2002;76(8):3784–90. 10.1128/JVI.76.8.3784-3790.2002 ; PubMed Central PMCID: PMCPMC136097. 11907218 PMC136097

[25] Nayak V, Dessau M, Kucera K, Anthony K, Ledizet M, Modis Y. Crystal structure of dengue virus type 1 envelope protein in the postfusion conformation and its implications for membrane fusion. J Virol. 2009;83(9):4338–44. 10.1128/JVI.02574-08 ; PubMed Central PMCID: PMCPMC2668458. 19244332 PMC2668458

[26] Kielian M, Rey FA. Virus membrane-fusion proteins: more than one way to make a hairpin. Nat Rev Microbiol. 2006;4(1):67–76. 10.1038/nrmicro1326 . 16357862 PMC7097298

[27] Hamamura Y, Saito C, Awai C, Kurihara D, Miyawaki A, Nakagawa T, et al Live-cell imaging reveals the dynamics of two sperm cells during double fertilization in Arabidopsis thaliana. Curr Biol. 2011;21(6):497–502. 10.1016/j.cub.2011.02.013 . 21396821

[28] von Besser K, Frank AC, Johnson MA, Preuss D. Arabidopsis HAP2 (GCS1) is a sperm-specific gene required for pollen tube guidance and fertilization. Development. 2006;133(23):4761–9. 10.1242/dev.02683 . 17079265

[29] Wong JL, Johnson MA. Is HAP2-GCS1 an ancestral gamete fusogen? Trends in cell biology. 2010;20(3):134–41. 10.1016/j.tcb.2009.12.007 . 20080406

[30] Chaw SM, Chang CC, Chen HL, Li WH. Dating the monocot-dicot divergence and the origin of core eudicots using whole chloroplast genomes. J Mol Evol. 2004;58(4):424–41. 10.1007/s00239-003-2564-9 . 15114421

[31] Lin C, Kuo FW, Chavanich S, Viyakarn V. Membrane lipid phase transition behavior of oocytes from three gorgonian corals in relation to chilling injury. PLoS ONE. 2014;9(3):e92812 10.1371/journal.pone.0092812 ; PubMed Central PMCID: PMC3966827. 24671092 PMC3966827

[32] Spricigo JF, Diogenes MN, Leme LO, Guimaraes AL, Muterlle CV, Silva BD, et al Effects of Different Maturation Systems on Bovine Oocyte Quality, Plasma Membrane Phospholipid Composition and Resistance to Vitrification and Warming. PLoS ONE. 2015;10(6):e0130164 10.1371/journal.pone.0130164 ; PubMed Central PMCID: PMC4480852. 26107169 PMC4480852

[33] Platre MP, Noack LC, Doumane M, Bayle V, Simon MLA, Maneta-Peyret L, et al A Combinatorial Lipid Code Shapes the Electrostatic Landscape of Plant Endomembranes. Dev Cell. 2018;45(4):465–80 e11. 10.1016/j.devcel.2018.04.011 . 29754803

[34] Angrisano F, Sala KA, Da DF, Liu Y, Pei J, Grishin NV, et al Targeting the Conserved Fusion Loop of HAP2 Inhibits the Transmission of Plasmodium berghei and falciparum. Cell Rep. 2017;21(10):2868–78. 10.1016/j.celrep.2017.11.024 ; PubMed Central PMCID: PMCPMC5732318. 29212032 PMC5732318

[35] Guardado-Calvo P, Rey FA. The Envelope Proteins of the Bunyavirales. Adv Virus Res. 2017;98:83–118. 10.1016/bs.aivir.2017.02.002 . 28433053

[36] Sprunck S, Rademacher S, Vogler F, Gheyselinck J, Grossniklaus U, Dresselhaus T. Egg cell-secreted EC1 triggers sperm cell activation during double fertilization. Science. 2012;338(6110):1093–7. 10.1126/science.1223944 . 23180860

[37] Valansi C, Moi D, Leikina E, Matveev E, Grana M, Chernomordik LV, et al Arabidopsis HAP2/GCS1 is a gamete fusion protein homologous to somatic and viral fusogens. J Cell Biol. 2017;216(3):571–81. 10.1083/jcb.201610093 ; PubMed Central PMCID: PMCPMC5350521. 28137780 PMC5350521

[38] Perez-Vargas J, Krey T, Valansi C, Avinoam O, Haouz A, Jamin M, et al Structural basis of eukaryotic cell-cell fusion. Cell. 2014;157(2):407–19. 10.1016/j.cell.2014.02.020 . 24725407

[39] Mori T, Kuroiwa H, Higashiyama T, Kuroiwa T. GENERATIVE CELL SPECIFIC 1 is essential for angiosperm fertilization. Nat Cell Biol. 2006;8(1):64–71. 10.1038/ncb1345 . 16378100

[40] Hirai M, Arai M, Mori T, Miyagishima SY, Kawai S, Kita K, et al Male fertility of malaria parasites is determined by GCS1, a plant-type reproduction factor. Curr Biol. 2008;18(8):607–13. 10.1016/j.cub.2008.03.045 . 18403203

[41] Kawai-Toyooka H, Mori T, Hamaji T, Suzuki M, Olson BJ, Uemura T, et al Sex-specific posttranslational regulation of the gamete fusogen GCS1 in the isogamous volvocine alga Gonium pectorale. Eukaryot Cell. 2014;13(5):648–56. 10.1128/EC.00330-13 ; PubMed Central PMCID: PMCPMC4060472. 24632243 PMC4060472

[42] Cole ES, Cassidy-Hanley D, Fricke Pinello J, Zeng H, Hsueh M, Kolbin D, et al Function of the Male-Gamete-Specific Fusion Protein HAP2 in a Seven-Sexed Ciliate. Current biology: CB. 2014;24(18):2168–73. 10.1016/j.cub.2014.07.064 . 25155508 PMC4313389

[43] Hernandez JM, Podbilewicz B. The hallmarks of cell-cell fusion. Development. 2017;144(24):4481–95. 10.1242/dev.155523 . 29254991

[44] Finn RD, Coggill P, Eberhardt RY, Eddy SR, Mistry J, Mitchell AL, et al The Pfam protein families database: towards a more sustainable future. Nucleic Acids Res. 2016;44(D1):D279–85. 10.1093/nar/gkv1344 ; PubMed Central PMCID: PMCPMC4702930. 26673716 PMC4702930

[45] Swanson WJ, Vacquier VD. The rapid evolution of reproductive proteins. Nat Rev Genet. 2002;3(2):137–44. 10.1038/nrg733 . 11836507

[46] Doms RW. What Came First-the Virus or the Egg? Cell. 2017;168(5):755–7. 10.1016/j.cell.2017.02.012 . 28235193

[47] Backovic M, Krey T. Stable Drosophila Cell Lines: An Alternative Approach to Exogenous Protein Expression. Methods in molecular biology (Clifton, NJ). 2016;1350:349–58. 10.1007/978-1-4939-3043-2_17 . 26820867

[48] Krey T, d'Alayer J, Kikuti CM, Saulnier A, Damier-Piolle L, Petitpas I, et al The disulfide bonds in glycoprotein E2 of hepatitis C virus reveal the tertiary organization of the molecule. PLoS Pathog. 2010;6(2):e1000762 10.1371/journal.ppat.1000762 ; PubMed Central PMCID: PMC2824758. 20174556 PMC2824758

[49] Kabsch W. Automatic indexing of rotation diffraction patterns. J Appl Crystallogr. 1988;(21):67–72.

[50] Evans P. Scaling and assessment of data quality. Acta Crystallogr D Biol Crystallogr. 2005;62(Pt 1):72–82. Epub 2005/12/22. doi: S0907444905036693 [pii] 10.1107/S0907444905036693 . 16369096

[51] Collaborative Computational Project. The CCP4 suite: programs for protein crystallography. Acta Crystallogr D Biol Crystallogr. 1994;50(Pt 5):760–3. Epub 1994/09/01. 10.1107/S0907444994003112 S0907444994003112 [pii]. . 15299374

[52] Bunkoczi G, Read RJ. Improvement of molecular-replacement models with Sculptor. Acta Crystallogr D Biol Crystallogr. 2011;67(Pt 4):303–12. 10.1107/S0907444910051218 ; PubMed Central PMCID: PMCPMC3069745. 21460448 PMC3069745

[53] McCoy AJ, Grosse-Kunstleve RW, Adams PD, Winn MD, Storoni LC, Read RJ. Phaser crystallographic software. J Appl Crystallogr. 2007;40(Pt 4):658–74. Epub 2007/08/01. 10.1107/S0021889807021206 ; PubMed Central PMCID: PMC2483472. 19461840 PMC2483472

[54] Thorn A, Sheldrick GM. ANODE: anomalous and heavy-atom density calculation. J Appl Crystallogr. 2011;44(Pt 6):1285–7. 10.1107/S0021889811041768 ; PubMed Central PMCID: PMCPMC3246834. 22477786 PMC3246834

[55] Emsley P, Lohkamp B, Scott WG, Cowtan K. Features and development of Coot. Acta Crystallogr D Biol Crystallogr. 2010;66(Pt 4):486–501. Epub 2010/04/13. doi: S0907444910007493 [pii] 10.1107/S0907444910007493 ; PubMed Central PMCID: PMC2852313. 20383002 PMC2852313

[56] Bricogne G, Blanc E, Brandl M, Flensburg C, Keller P, Paciorek P, et al. BUSTER version 2.9 [software]. Cambridge, United Kingdom, Global Phasing. 2010 [cited 2016 August 16th].

[57] Hellens RP, Edwards EA, Leyland NR, Bean S, Mullineaux PM. pGreen: a versatile and flexible binary Ti vector for Agrobacterium-mediated plant transformation. Plant Mol Biol. 2000;42(6):819–32. . 10890530 10.1023/a:1006496308160

[58] Koncz C, Schell J. The Promoter of Tl-DNA Gene 5 Controls the Tissue-Specific Expression of Chimeric Genes Carried by a Novel Type of Agrobacterium Binary Vector. Molecular & General Genetics. 1986;204(3):383–96. PubMed PMID: ISI:A1986D929100004.

[59] Alonso JM, Stepanova AN, Leisse TJ, Kim CJ, Chen H, Shinn P, et al Genome-wide insertional mutagenesis of Arabidopsis thaliana. Science. 2003;301(5633):653–7. 10.1126/science.1086391 . 12893945

[60] Clough SJ, Bent AF. Floral dip: a simplified method for Agrobacterium-mediated transformation of *Arabidopsis thaliana*. Plant J. 1998;16(6):735–43. . 10069079 10.1046/j.1365-313x.1998.00343.x

[61] Celenza JL Jr., Grisafi PL, Fink GR. A pathway for lateral root formation in *Arabidopsis thaliana*. Genes & development. 1995;9:2131–42. 7657165 10.1101/gad.9.17.2131

[62] Wilson ZA, Morroll SM, Dawson J, Swarup R, Tighe PJ. The Arabidopsis *MALE STERILITY1 (MS1)* gene is a transcriptional regulator of male gametogenesis, with homology to the PHD-finger family of transcription factors. Plant Journal. 2001;28(1):27–39. PubMed PMID: ISI:000171765000003. 11696184 10.1046/j.1365-313x.2001.01125.x

[63] Boavida LC, McCormick S. Temperature as a determinant factor for increased and reproducible in vitro pollen germination in Arabidopsis thaliana. Plant J. 2007;52(3):570–82. 10.1111/j.1365-313X.2007.03248.x . 17764500

[64] Schindelin J, Arganda-Carreras I, Frise E, Kaynig V, Longair M, Pietzsch T, et al Fiji: an open-source platform for biological-image analysis. Nat Methods. 2012;9(7):676–82. 10.1038/nmeth.2019 ; PubMed Central PMCID: PMCPMC3855844. 22743772 PMC3855844

[65] RStudio Team. RStudio: Integrated Development for R. RStudio. Boston, MA: RStudio; 2016 Available from: http://www.rstudio.com

[66] R Core Team. R: A language and environment for statistical computing. Vienna, Austria: R Foundation for Statistical Computing; 2015 Available from: https://www.R-project.org

[67] Baldauf SL, Roger AJ, Wenk-Siefert I, Doolittle WF. A kingdom-level phylogeny of eukaryotes based on combined protein data. Science. 2000;290(5493):972–7. . 11062127 10.1126/science.290.5493.972

[68] Csuros M, Rogozin IB, Koonin EV. A detailed history of intron-rich eukaryotic ancestors inferred from a global survey of 100 complete genomes. PLoS Comput Biol. 2011;7(9):e1002150 10.1371/journal.pcbi.1002150 ; PubMed Central PMCID: PMC3174169. 21935348 PMC3174169

[69] Krogh A, Larsson B, von Heijne G, Sonnhammer EL. Predicting transmembrane protein topology with a hidden Markov model: application to complete genomes. J Mol Biol. 2001;305(3):567–80. 10.1006/jmbi.2000.4315 . 11152613

[70] Juretic D, Zoranic L, Zucic D. Basic charge clusters and predictions of membrane protein topology. J Chem Inf Comput Sci. 2002;42(3):620–32. . 12086524 10.1021/ci010263s

[71] Ebchuqin E, Yokota N, Yamada L, Yasuoka Y, Akasaka M, Arakawa M, et al Evidence for participation of GCS1 in fertilization of the starlet sea anemone Nematostella vectensis: implication of a common mechanism of sperm-egg fusion in plants and animals. Biochem Biophys Res Commun. 2014;451(4):522–8. 10.1016/j.bbrc.2014.08.006 . 25111819

