# Supplementary figures and images for: Evolutionary diversification of the HAP2 membrane insertion motifs to drive gamete fusion across eukaryotes
Source: PLoS Biol. 2018 Aug 13;16(8):e2006357. doi: 10.1371/journal.pbio.2006357 (PMC6089408; doi:10.1371/journal.pbio.2006357)

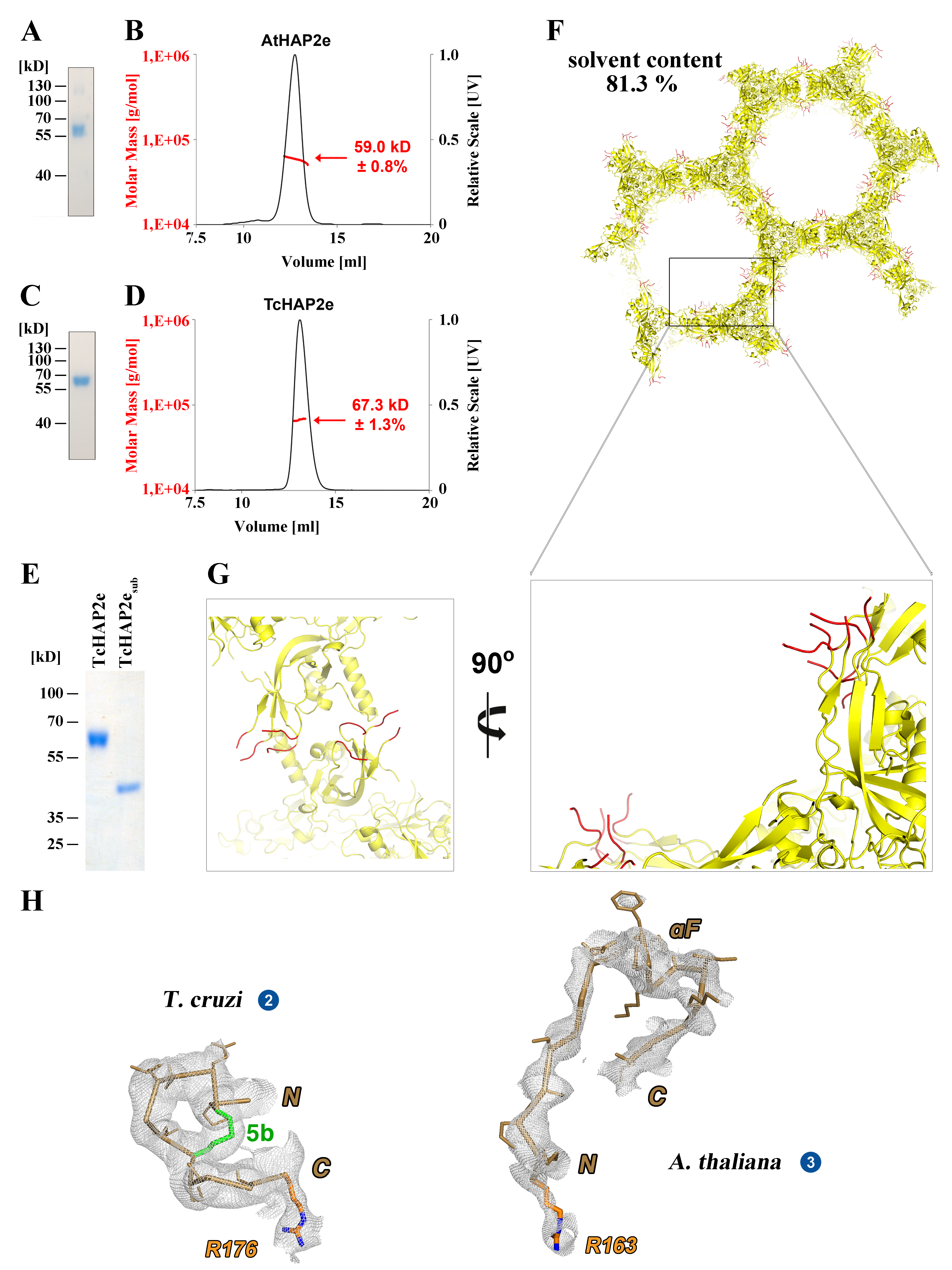

Supplement: S1 Fig — (A) AtHAP2e and (C) TcHAP2e purity was assessed by Coomassie blue–stained SDS-PAGE. SEC analysis of AtHAP2e (C) and (D) TcHAP2e on a Superdex 200 column in 10 mM Tris pH8, 100 mM NaCl revealed the presence of a single peak. SEC-MALS analysis indicated that this peak for both Tc and AtHAP2e corresponds to a monomer. The y-axis on the right gives the UV absorption at 280 nm on a relative scale from 0 to 1 (plotted in black), while the y-axis on the left gives the molecular mass by MALS (plotted and labeled in red). (E) TcHAP2e and the purified protease-resistant fragment resulting from subtilisin cleavage (TcHAP2esub) were separated by SDS-PAGE stained with Coomassie blue. (F+G) Overview (top panel) and close-up (bottom panel) of the TcHAP2esub crystal packing. Domain II (yellow) assembles as a rigid dodecameric ring, whereas domain I projects into solvent within this ring, explaining why domain I is only partially traceable in the experimental electron density. (H) Composite omit electron density of part of the cd strand connection of TcHAP2 (left) and AtHAP2 (right) corresponding to variable regions 2 and 3, respectively, contoured at 0.9σ, allowing for manual building of the model in this region. AtHAP2e, A. thaliana HAPLESS 2 ectodomain; MALS, multiangle static light scattering; SEC, size exclusion chromatography; TcHAP2e, T. cruzi HAPLESS 2 ectodomain. (TIF) [file pbio.2006357.s001.tif]

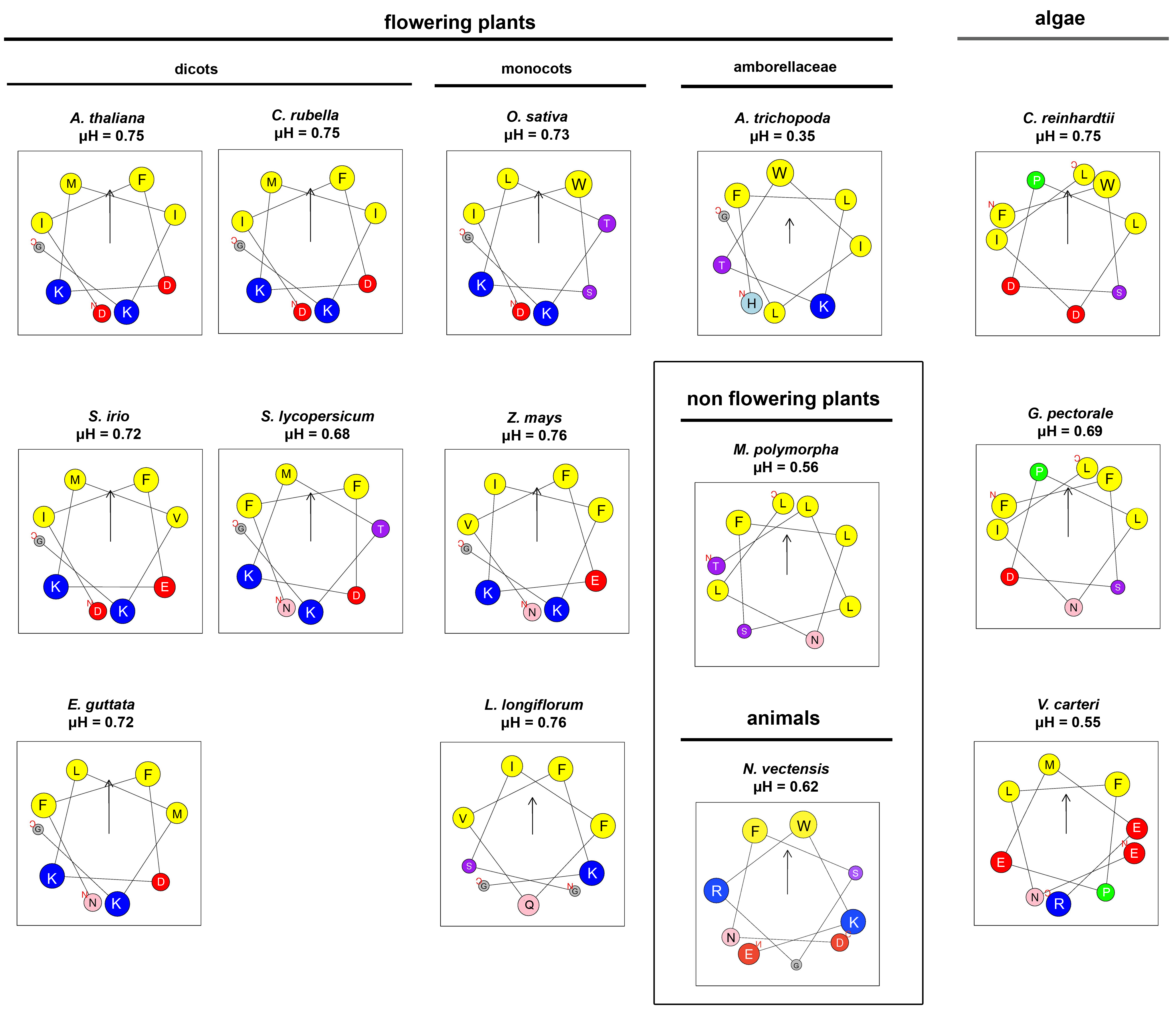

Supplement: S2 Fig — Helical wheel projections of HAP2 loop 2 residues from indicated organisms. The circle size is proportional to the size of the displayed side chain. Nonpolar residues are colored in yellow. Charged residues are colored red (negative charge) and blue (positive charge). Polar uncharged residues are purple and light pink. Glycines are displayed in gray, prolines in green. The hydrophobic moment value of the respective amphipathic helix predicted by Heliquest (μH) is shown below the organism name, suggesting the formation of an amphipathic helix in the putative membrane-interacting region of plant and algal HAP2 but also in some animal HAP2 orthologs. HAP2, HAPLESS 2. (TIF) [file pbio.2006357.s002.tif]

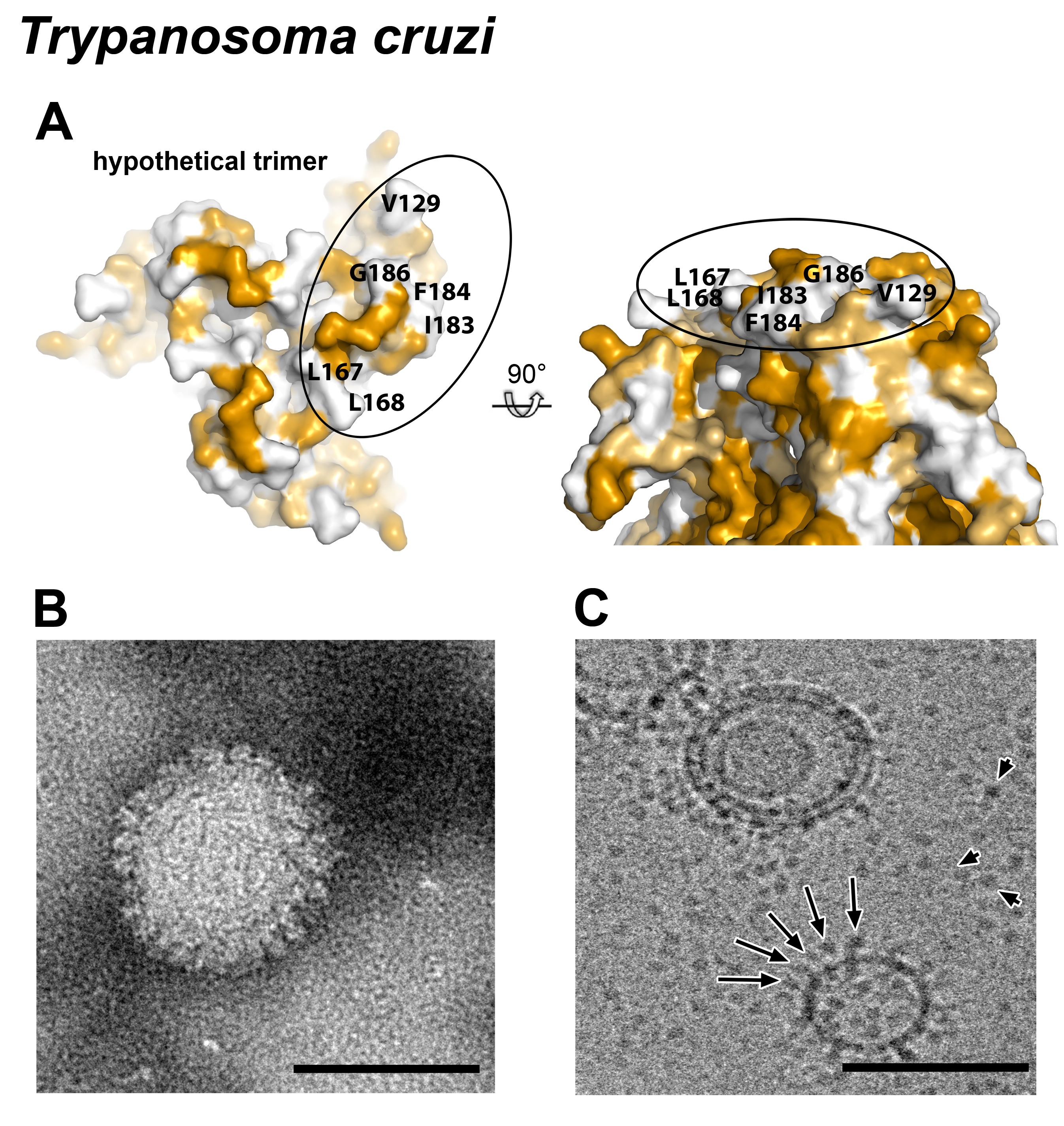

Supplement: S3 Fig — (A) Surface representation of the membrane-facing tip of the putative TcHAP2 trimer, obtained as described in Materials and methods, viewed from the membrane (left panel) and from the side (right panel). Surface residues are colored according to hydrophilicity from dark orange (hydrophilic) to white (hydrophobic). The fusion loop region from one protomer is encircled, and hydrophobic residues that are exposed at the tip are labeled. Residue 129 belongs to the bc connection, the others to cd. (B-C) Membrane insertion of TcHAP2e. Electron micrographs of liposomes incubated in presence of HAP2e were analyzed by negative staining (B) or cryo-EM (C). Scale bar 50 nm. Liposomes decorated with HAP2e display protein projections at the surface. The protein projections (some of which are indicated by long arrows in C) are shaped as tapered rods similar to the ones of AtHAP2e (see Fig 4) and form lateral assemblies. The background is coated with proteins not bound to liposomes (short arrows in C). AtHAP2e, A. thaliana HAPLESS 2 ectodomain; EM, electron microscopy; TcHAP2, T. cruzi HAPLESS 2; TcHAP2e, T. cruzi HAPLESS 2 ectodomain. (TIF) [file pbio.2006357.s003.tif]

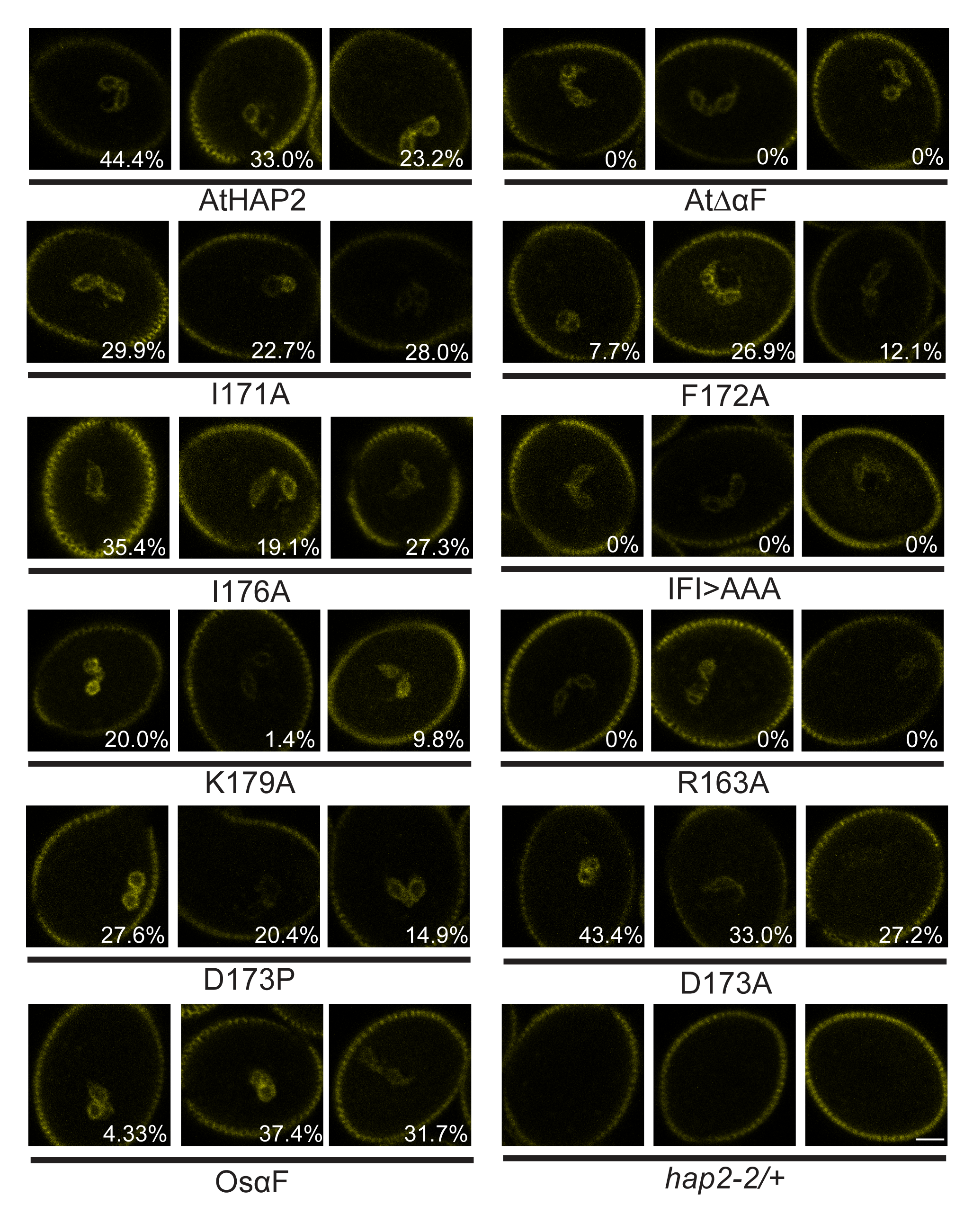

Supplement: S4 Fig — Confocal micrographs of pollen grains from three independent transgenic lines expressing the indicated HAP2:YFP variant construct are shown. Each pollen grain shown contains a pair of sperm representing the maximum YFP intensity observed in the represented transgenic line. Signal from the pollen grain wall is due to autofluorescence and observed in the hap2-2/+ control, which does not contain a HAP2:YFP construct. S4 Table lists the number of independent transgenic lines that were tested for each HAP2:YFP variant using our genetic transmission assay for HAP2 function (Fig 6). The average percent transmission of hap2-2 (33% is expected for a fully functional single-insertion-site line) for each line is indicated in the bottom-right corner of each image. Scale bar, 5 μm. HAP2, HAPLESS 2; YFP, yellow fluorescent protein. (TIF) [file pbio.2006357.s004.tif]

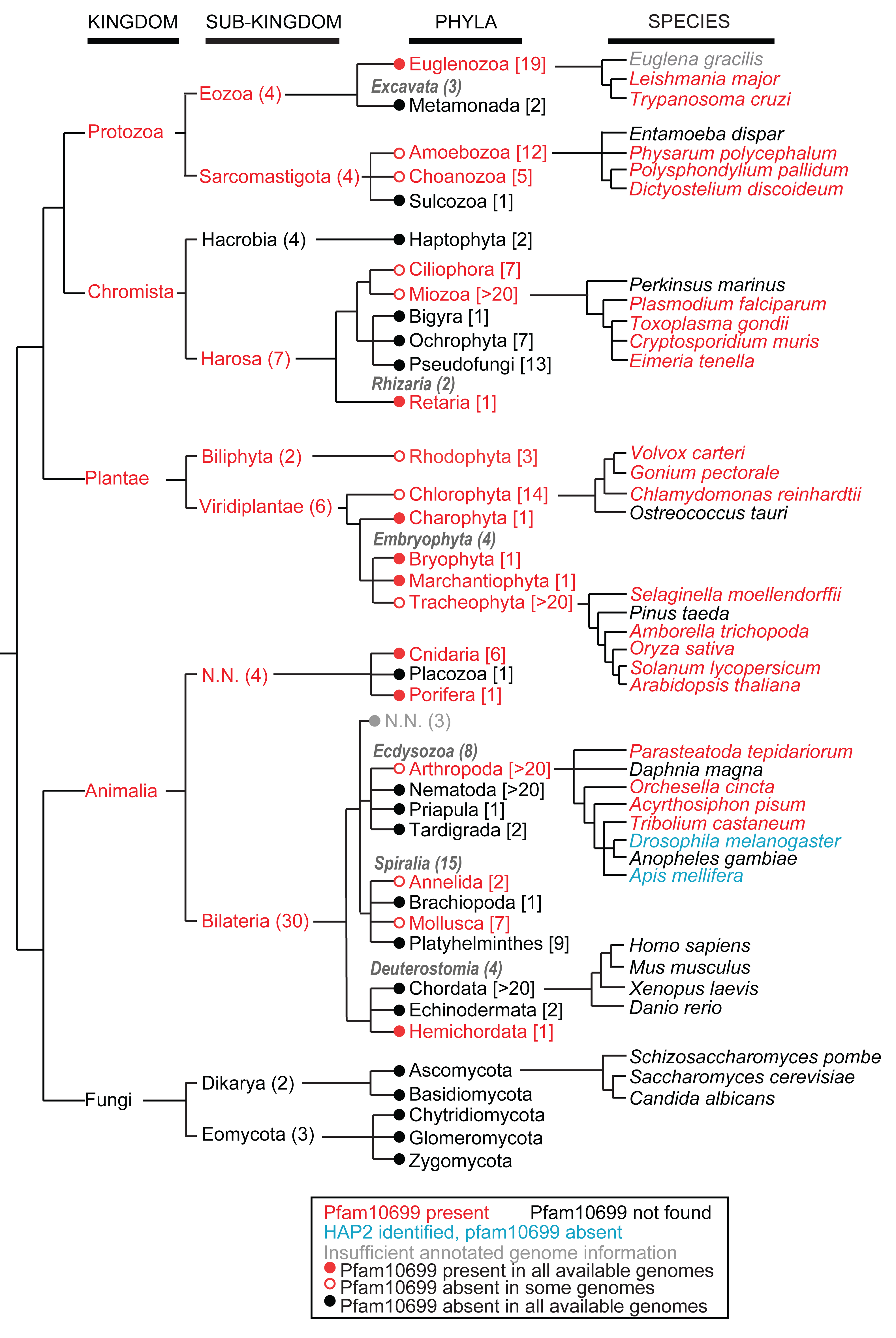

Supplement: S5 Fig — A schematic phylogeny representing the five eukaryotic kingdoms, subkingdoms, and phyla for which whole-genome sequence information is available (classification system; [17, 67]). Selected groups of species are provided for some phyla. Red font indicates presence of pfam10699 (the HAP2-GCS1 domain). Filled red circles: all available genomes of the phylum contain pfam10699; open red circles: the phylum contains examples both of genomes that have or do not have pfam10699; filled black circles: phyla in which none of the available genomes contains pfam10699. HAP2 was identified in D. melanogaster and Apis mellifera [4], but these sequences (and genomes) lack pfam10699 (blue font). Parenthetical numbers indicate the total number phyla within the subkingdom. Only phyla with at least one whole-genome sequence with >10,000 annotated proteins are listed. Bracketed numbers indicate the number of species from each phylum used in this analysis. GCS1, GENERARATIVE CELL SPECIFIC 1; HAP2, HAPLESS 2. (TIF) [file pbio.2006357.s005.tif]

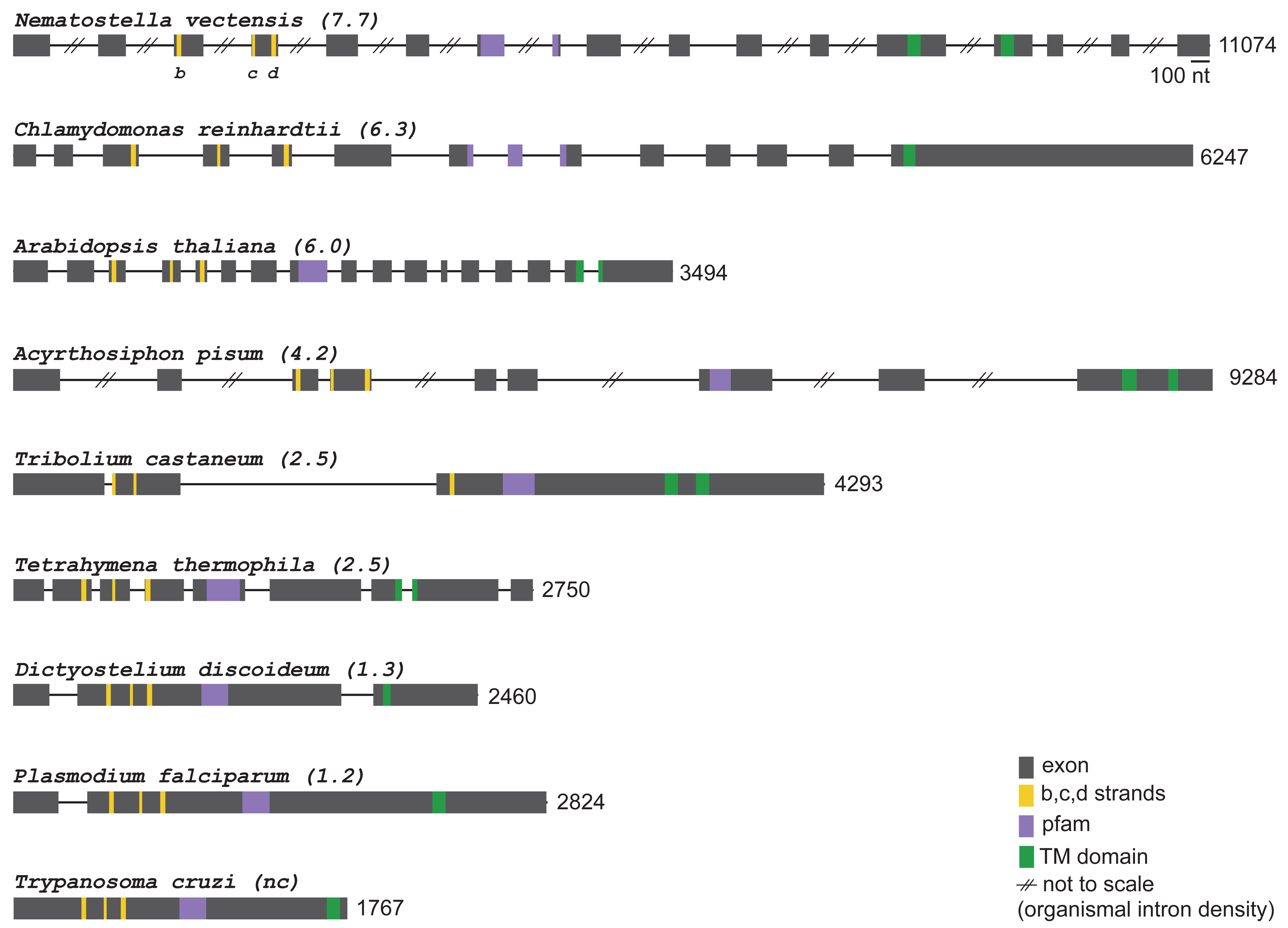

Supplement: S6 Fig — The HAP2 gene organization from organisms representing four eukaryotic kingdoms (Plantae: A. thaliana, C. reinhardtii; Chromista: Plasmodium falciparum, T. thermophila; Protozoa: T. cruzi, Dictyostelium discoideum; Animalia, N. vectensis, T. castaneum, Acyrthosiphon pisum; S1 Table). Sequences have been ordered by decreasing organismal intron density (introns/1-kb coding sequence [68], in parentheses next to species name). The T. cruzi genome contains only a few introns, so intron density was not calculated (“nc”, [68]). Segments encoding the bdc β-sheet are highlighted (yellow) together with the location of the pfam10699 segment (purple) and the TM segments (green). TMs were identified from Uniprot for C. reinhardtii (A4GCR6.2), A. thaliana (F4JP36.1), and T. thermophila (A0A060A682) or by prediction (TMHMM server [69] or the Split 4.0 server [70]). TM predictions for N. vectensis were previously published [71]. Note that the β-strands b, c, and d are only encoded by one single exon in organisms with very few introns, suggesting a potential mode of evolution via focal variation at the interstrand connections. HAP2, HAPLESS 2; TM, transmembrane. (TIF) [file pbio.2006357.s006.tif]
