# Supplementary material for: Evolutionary diversification of the HAP2 membrane insertion motifs to drive gamete fusion across eukaryotes
Source: PLoS Biol. 2018 Aug 13;16(8):e2006357. doi: 10.1371/journal.pbio.2006357 (PMC6089408; doi:10.1371/journal.pbio.2006357)
Supplement: S1 Table — (PDF) [file pbio.2006357.s007.pdf]

Table S1. Accession numbers of sequences used in this study.

Organism	Protein accession	mRNA/cds	genomic
Chlamydomonas reinhardtii	A4GRC6.2[1]	EF397563.2	Cre16.g674852[2]
Gonium pectorale	BAO57179.1	AB915401.1	AB915402.1
Volvox carteri	EFJ46133.1	-----	GL378353.1
Cyanidioschyzon merolae	XP_005536505.1	XM_005536448. 1	NC_010137.1; CYME_CMK076C
Selaginella moellendorfii	XP_002971451.1	XM_002971405.1 (partial)	NW_003314278.1; SELMODRAFT_451371; GCS1-2
Physcomitrella patens	*	*	Pp3c25_15230[2] (partial)*
Marchantia polymorpha	BAV38815.1	LC172179.1	Mapoly0066s0032[2]
Arabidopsis thaliana	AAY51999.1	DQ022375.1	NC_003075.7; HAP2
Capsella rubella	XP_006289435.1	XM_006289373.1	NW_006238918.1; CARUB_v10002940mg
Sisymbrium irio	ADE20442.1	GU724984.1 (partial)	-----
Erythranthe guttata	XP_012846387.1	XM_012990933.1	NW_012193798.1; LOC105966372
Solanum lycopersicum	XP_019070618.1	XM_019215073.1	NC_015445.2; LOC101265357
Lilium longiflorum	BAE71142.1	AB206810.1	-----
Zea mays	NP_001307741.1	NM_001320812.1	NC_024460.2; LOC100274016
Oryza sativa	BAF16968.1	AK072871.1	LOC_Os05g18730[2]
Amborella trichopoda	XP_020531486.1	XM_020675827.1	NW_006500346.1; LOC18447729
Toxoplasma gondii	KYF43852.1	-----	AGQS02004567.1
Cryptosporidium muris	EEA05614.1	XM_002139927.1	NW_002196571.1; CMU_026210
Plasmodium falciparum	XP_001347424.1	PF3D7_1014200[3]	PF3D7_1014200; NC_037281.1
Eimeria tenella	BAM16295.1	AB723702.1	ETH_00017050/ETH_00017055[4,5]
Tetrahymena thermophila	AIA57699.1	KJ629172.1	GG662480.1; NW_002476194.1; TTHERM_01075640
Paramecium tetraurelia	CAK65033.1	XM_001432393.1 (partial)	NW_001799006.1; GSPATT00034511001
Trypanosoma cruzi	EAN93043.1	XM_809801.1 (partial)	NW_001849404.1; Tc00.1047053509105.4
Trypanosoma congolense	CCC94129.1	-----	HE575323.1
Trypanosoma	EAN78468.1	-----	CM000208.1

brucei			
Leishmania major	XP_003722443.1	XM_003722395.1 (partial)	NC_007284.2; LMJF_35_0460
Naegleria gruberi	EFC41606.1	XM_002674304.1	NW_003163287.1; NAEGRDRAFT_70404
Dictyostelium discoideum	BAS29571.1	LC075766.1	DDB_G0276069[6]
Physarum polycephalum	BAE71144.1	AB206812.1	-----
Monosiga brevicolis	EDQ88884.1	XM_001746445.1	NW_001865049.1; MONBRDRAFT_8819
Amphimedon queenslandica	XP_019853252.1	XM_019997693.1	NW_003546408.1; LOC109582771
Nematostella vectensis	EDO36432.1 XP_001628495	XM_001628445.1 (partial)	NW_001834250.1; NEMVEDRAFT_v1g212848
Hydra vulgaris	XP_004211319.2	XM_004211271.2	NW_004172600.1; LOC101240634
Capitella teleta	ELU07639.1	-----	KB299712.1
Tribolium castaneum	EFA06462.1	XM_008196950.2	NC_007422.5; LOC103313508
Acyrthosiphon pisum	XP_016661643.1	XM_016806154.1	NW_003384018.1; LOC100569456
Orchesella cincta	ODN05384.1	-----	LJIJ01000024.1
Saccoglossus kowalevskii	XP_006821859.1	LOC102804021	NW_003142801; LOC102804021

[1]UniProtKb/Swiss-Prot ID in NCBI. [2]Phytozome ID. [3]PlasmoDB ID. [4]ToxoDB. [5]Walker et al. 2015. [6]DictyBase ID.
*Personal communication with A.-C. Lindner and J. Becker: includes analysis of RNA-seq data and protein annotation.
