# Supplementary material for: Evolutionary diversification of the HAP2 membrane insertion motifs to drive gamete fusion across eukaryotes
Source: PLoS Biol. 2018 Aug 13;16(8):e2006357. doi: 10.1371/journal.pbio.2006357 (PMC6089408; doi:10.1371/journal.pbio.2006357)
Supplement: S2 Table — (PDF) [file pbio.2006357.s008.pdf]

Table S2. Data collection, phasing and refinement statistics.

	AtHAP2		TcHAP2
**Data collection**			
Space group	*P* 6_3		*P* 6_1 22
Cell dimensions			
a, *b*, *c* (Å)	77.26 77.26 219.53		150.10 150.10 123.80
α, *β*, *γ* (°)	90.0 90.0 120.0		90.0 90.0 120.0
Resolution (Å)	49.33-2.75 (2.82-2.75)[a]	49.33-2.75 (2.82-2.75)[b]	49.12-3.1 (3.31-3.1)
R_{meas}	0.256 (6.2)	0.091 (1.05)	0.163 (1.74)
I/σ (I)	6.08 (0.37)	8.58 (0.95)	11.4 (1.3)
$CC_{1/2}$	0.998 (0.36)	0.997 (0.40)	0.997 (0.41)
Completeness (%)	99.7 (99.2)	51.9 (25.2)	97.7 (98.2)
Redundancy	7.0 (6.9)	3.2 (2.4)	8.9 (8.6)
**Refinement**			
Resolution (Å)	49.38-2.75 (3.07-2.75)		49.12-3.1 (3.31-3.1)
No. reflections	10646		15430
R_{work} / R_{free}	0.231 / 0.277		0.233 / 0.26
No. atoms			
Protein	3511		1855
Solvent	43		2
B factors			
Protein	67.80		84.33
R.m.s deviations			
Bond lengths (Å)	0.007		0.008
Bond angles (°)	0.91		0.99
Ramachandran plot[§]			
Favoured (%)	94.1		96.1
Allowed (%)	5.9		3.9
Outliers (%)	0		0

One crystal was used to collect each of the diffraction data sets used to determine the crystal structure.

[a] Values in parentheses are for highest-resolution shell.
[b] After ellipsoidic truncation performed using the StarAniso server (Global Phasing Ltd.).
[§] Ramachandran statistics were calculated with MolProbity
