# Supplementary material for: Evolutionary diversification of the HAP2 membrane insertion motifs to drive gamete fusion across eukaryotes
Source: PLoS Biol. 2018 Aug 13;16(8):e2006357. doi: 10.1371/journal.pbio.2006357 (PMC6089408; doi:10.1371/journal.pbio.2006357)
Supplement: S3 Table — (PDF) [file pbio.2006357.s009.pdf]

Table S3. Structural similarity across class II fusion proteins.

	***Cr*HAP2**	***At*HAP2**	***Tc*HAP2**	RV	TBEV	SFV	RVFV	EFF1
***Cr*HAP2**		z=20.1<br>rmsd=2.8<br>210/234<br>32 %id	**z=21.9<br>rmsd=2.0<br>209/237<br>34 %id**	**z=3.2<br>rmsd=3.9<br>130/276<br>8 %id**	**z=5.5<br>rmsd=5.5<br>150/198<br>5 %id**	**z=5.9<br>rmsd=4.7<br>152/212<br>11 %id**	**z=5.8<br>rmsd=4.3<br>145/217<br>7 %id**	**z=6.7<br>rmsd=4.9<br>159/242<br>6 %id**
***At*HAP2**			z=18.5<br>rmsd=3.1A<br>215/237<br>28 %id	**z=3.7<br>rmsd=4.2<br>149/276<br>5 %id**	**z=5.1<br>rmsd=3.7<br>94/198<br>6 %id**	**z=6.2<br>rmsd=3.8<br>118/212<br>12 %id**	**z=5.9<br>rmsd=5.0<br>149/217<br>7 %id**	**z=6.5<br>rmsd=4.5<br>157/242<br>8 %id**
***Tc*HAP2**				**z=2.9<br>rmsd=3.9<br>119/276<br>6 %id**	**z=5.2<br>rmsd=5.2<br>139/198<br>4 %id**	**z=5.4<br>rmsd=5.0<br>153/212<br>7 %id**	**z=5.5<br>rmsd=4.3<br>143/217<br>7 %id**	**z=6.7<br>rmsd=4.2<br>160/242<br>9 %id**
RV					**z=5.6<br>rmsd=4.4<br>108/198<br>3 %id**	**z=5.9<br>rmsd=6.3<br>164/212<br>6 %id**	**z=6.1<br>rmsd=5.4<br>164/217<br>4 %id**	**z=5.6<br>rmsd=6.0<br>171/242<br>8 %id**
TBEV						z=9.1<br>rmsd=3.7<br>180/212<br>8 %id	z=8.7<br>rmsd=4.3<br>169/217<br>10 %id	z=7.3<br>rmsd=5.8<br>172/242<br>8 %id
SFV							z=9.6<br>rmsd=4.2<br>172/217<br>11 %id	z=9.3<br>rmsd=6.0<br>189/242<br>9 %id
RVFV								z=9.6<br>rmsd=4.0<br>184/242<br>9 %id
EFF1								

Dali score color-code:

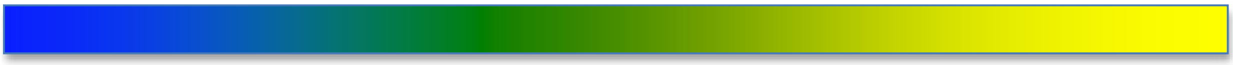

[a]Statistics from a pairwise analysis with the DALI server [15] focusing on domain II from the class II fusion proteins listed. The background is color-coded according to the DALI score, as in the bar underneath. For reference, Z scores below 2 are meaningless, whereas values of around 50 are obtained when structures of the same protein from two different crystal forms are compared (serving as reference for proteins of roughly the same size). rmsd is the root mean square deviation between Cα atoms (in Å). The third line in each box is "N/N^T", where N is the number of aligned residues (N) compared to the total residues in the alignment (N^T). "%id" indicates % amino acid identity after the alignment.
