# Supplementary material for: Evolutionary diversification of the HAP2 membrane insertion motifs to drive gamete fusion across eukaryotes
Source: PLoS Biol. 2018 Aug 13;16(8):e2006357. doi: 10.1371/journal.pbio.2006357 (PMC6089408; doi:10.1371/journal.pbio.2006357)
Supplement: S4 Table — HAP2, HAPLESS 2; YFP, yellow fluorescent protein. (PDF) [file pbio.2006357.s010.pdf]

Table S4. Transgenic lines used to analyze function of HAP2:YFP variants.

Construct ID	HAP2 Variant	Lines (n)	Crosses (n)	Seedlings / Cross (avg.)	Progeny (n)
pgl294	AtHAP2	8	39	41.9	1635
pgl1070	AtΔαF	6	28	29.5	825
pgl1165	I171A	5	33	43.9	1448
pgl1061	F172A	7	44	34.4	1513
pgl1057	I176A	8	46	41.3	1900
pgl1315	IFI>AAA	6	26	24.4	634
pgl1371	R163A	4	24	30.0	721
pgl1510	D173P	8	47	46.0	2164
pgl1374	D173A	7	39	38.6	1507
pgl1056	K179A	7	33	36.2	1195
pgl1151	OsαF	9	46	34.6	1592
	hap2-2/HAP2	n/a	39	31.9	1244

Total numbers (n) of independent transgenic lines (Lines), cross-pollinations (Crosses), progeny tested (Progeny), and the average number of seedlings analyzed for each cross pollination (Seedlings/Cross) are given for each construct. ID, construct identification number
