# Supplementary material for: Evolutionary diversification of the HAP2 membrane insertion motifs to drive gamete fusion across eukaryotes
Source: PLoS Biol. 2018 Aug 13;16(8):e2006357. doi: 10.1371/journal.pbio.2006357 (PMC6089408; doi:10.1371/journal.pbio.2006357)
Supplement: S5 Table — HAP2, HAPLESS 2; YFP, yellow fluorescent protein. (PDF) [file pbio.2006357.s011.pdf]

Table S5. Primers used for generation of HAP2:YFP variants and for genotyping and sequencing transformed plants.

Primer Name	Primer	Reason
hap2SeqTR3	CAATCAAACTGCGCAGAAGGAAGC	*hap2-2* genotyping
Hap2C2	GGCCTCACTCGTTCTCAATTGGAG	*hap2-2* genotyping
Lba1	TGGTTCACGTAGTGGGCCATCG	*hap2-2* genotyping
HAP2Ex7R	GATTAACTTTGAGAAAGTTATCATTTG	sequencing variants
ECORI_HAP2_CVFP_F1	gaattcCTTACTTACATTAGGGATGTACCT	sequencing variants
HAP2_FL_F172A_F1	GTGGAGATATTGCTGATAAGATG	F172A
HAP2_FL_F172A_R1	AAGAAGAAGGCATTCTTCGTT	F172A
HAP2_FL_K179A_F1	GATTAAAGGGGCGGCAAATACC	K179A
HAP2_FL_K179A_R1	ATCTTATCAAAAATATCTCCACAAGAAGAAGGC	K179A
HAP2_FL_I176A_F1	TGATAAGATGGCTAAAGGGAAG	I176A
HAP2_FL_I176A_R1	AAAATATCTCCACAAGAAGAAGGC	I176A
HAP2_del_pFL_F1	GCAAATACCGCGCATTGCCTTCG	Δ169-179
HAP2_del_pFL_R1	ACAAGAAGAAGGCATTCTTCGTTGCGGTCC	Δ169-179
HAP2_OspFL_R1	CTTACTCCAAATATCTCCACAAGAAGAAGGCAT	Os.FH replacement
HAP2_OspFL_F1	TTGACAAAAGGGAAGGCAAATACCGCGCA	Os.FH replacement
HAP2_I171A_triple_R1	AAGAAGGCATTCTTCGTTGCGGTC	I171A
HAP2_I171A_F2	CTTGTGGAGATGCTTTTGATAAGAT	I171A

HAP2_triple_build_R2	CTTATCAGCAGCATCTCCACAAGAAGAAGGCATTC	IFI>AAA
HAP2_triple_build_F2	ATGGCTAAAGGGAAGGCAAATACCGCGCATTG	IFI>AAA
R185A_AtHAP2_R1	CACATGGACAACAAATCGGCTGAGTC	R163A
R185A_AtHAP2_F1	GACCGCAACGAGCAATGCCTTCTTCT	R163A
HAP2_D173A_F1	GAGATATTTTTGCTAAGATGATTAAAGGGAAGGC	D173A
HAP2_D173A_R1	CACAAGAAGAAGGCATTCTTCGTTGC	D173A
HAP2_D173P_F1	GTGGAGATATTTTTCCCAAGATGATTAAAG	D173P
HAP2_D173P_R1	AAGAAGAAGGCATTCTTCGTT	D173P
